# Supplementary material for: One-year recovery rates for young people with depression and/or anxiety not receiving treatment: a systematic review and meta-analysis
Source: BMJ Open. 2023 Jul 21;13(7):e072093. doi: 10.1136/bmjopen-2023-072093 (PMC10364186; doi:10.1136/bmjopen-2023-072093)
Supplement: Supplementary data [file bmjopen-2023-072093supp002.pdf]

NIHR | National Institute for Health Research

PROSPERO
International prospective register of systematic reviews

One year outcomes for young people with depression and/or anxiety: a systematic review

Citation

Anna Roach, Diliniya Sureshkumar, Stefan Priebe. One year outcomes for young people with depression and/or anxiety: a systematic review. PROSPERO 2021 CRD42021251556 Available from: https://www.crd.york.ac.uk/prospero/display_record.php?ID=CRD42021251556

Review question

What are the one year outcomes for young people with depression and/or anxiety?

Searches

Five databases will be searched: MEDLINE via PubMed, PsycINFO, Web of Science, Embase, Global Health.

MeSH terms to be used: "depression" "anxiety" "emotions" "psychological distress" "adolescent".

Inclusion:

• Longitudinal cohort research studies (with one year follow-up data);

• Texts must include primary data on anxiety and/or depression.

Exclusion:

• Studies of a specific clinical intervention/treatment;

• Grey literature (due to lack of peer-review).

The search will be re-run prior to submission.

Types of study to be included

Inclusion:

• Longitudinal cohort research studies (with one year follow-up data);

• Texts must include primary data on anxiety and/or depression.

Studies from clinical and non-clinical contexts will be included.

Research from any country will be included.

Exclusion:

• Studies of a specific clinical intervention/treatment;

• Grey literature (due to lack of peer-review).

Condition or domain being studied

Depression and/or anxiety in young people.

Participants/population

Inclusion:

- Participants defined by WHO as "young people" (aged 10-24) at baseline. This includes adolescents (aged 10-19) and youth (aged 15-24);

- Young people with baseline symptom levels above a specified clinical cut off point and/or a diagnosis of depression and/or anxiety as defined by the ICD-10 or equivalent. This will be indicated by a validated self-report or observer-rated questionnaire or interview.

Exclusion:

- None.

Intervention(s), exposure(s)

The focus of this review is assessing one year outcomes for young people experiencing depression and/or anxiety.

Comparator(s)/control

Not applicable.

Context

Main outcome(s)

Main outcome: recovery rate.

Secondary outcome: symptom level change.

Additional outcome(s)

Measures of quality of life and school and work performance, and social functioning will be included in our analysis of secondary outcomes.

Data extraction (selection and coding)

A minimum of two researchers will be involved in the screening, appraisal, and data extraction. Titles and abstracts will initially be screened by one author and duplicates removed. A second review author will independently screen 25% of these to check for consistency.

The full texts of potentially eligible studies will then be retrieved and assessed for eligibility by one author, and a second researcher will screen a random subset of 25% of potentially eligible studies. Any disagreements will be resolved by discussion with a third author.

MS Excel will be used to create a data extraction table. Extracted information will include study objective,

Supplemental material BMJ Publishing Group Limited (BMJ) disclaims all liability and responsibility arising from any reliance placed on this supplemental material which has been supplied by the author(s) BMJ Open

country/setting of research, sample size, baseline outcome measure, validated measure of depressive and/or anxious symptoms (including if self-report/clinician led), age at baseline, follow up duration, follow up outcome, method of analysis, covariates and notes.

Risk of bias (quality) assessment

Quality assessment will be conducted by a minimum of two review authors alongside data extraction. In order to ensure that both researchers are assessing the quality of included texts reliably, 25% of the papers will initially be extracted by both reviewers and then compared. Any inconsistencies will be thoroughly discussed. For each study, researchers will use the Newcastle-Ottawa Scale to assess methodological quality.

Strategy for data synthesis

Study characteristics will be summarised in text, and provided in table format.

Meta-analysis will be conducted if studies are similar enough to pool (through assessments for clinical and statistical heterogeneity), and there are a sufficient number of studies using similar outcome measures. If not, a narrative synthesis will be conducted.

Analysis of subgroups or subsets

If a large enough subsample is identified, subgroup analysis of different age groups may be carried out. We will consider analysis of gender, race, country of study, different types of samples e.g. college students, and symptom level at baseline.

Contact details for further information

Anna Roach

anna.roach3@nhs.net

Organisational affiliation of the review

Unit for Social and Community Psychiatry (World Health Organisation Collaborating Centre for Mental Health Service Development), Newham Centre for Mental Health, London E13 8SP

https://www.qmul.ac.uk/socialpsych/

Review team members and their organisational affiliations

Miss Anna Roach. East London NHS Trust

Ms Diliniya Sureshkumar. Unit for Social and Community Psychiatry, Queen Mary University of London

Professor Stefan Priebe. Unit for Social and Community Psychiatry, Queen Mary University of London

Type and method of review

Epidemiologic, Prognostic, Systematic review

Anticipated or actual start date

03 May 2021

Anticipated completion date

Roach A, *et al. BMJ Open* 2023; 13:e072093. doi: 10.1136/bmjopen-2023-072093

31 August 2021

Funding sources/sponsors

Own account

Conflicts of interest

Language

English

Country

England

Stage of review

Review Ongoing

Subject index terms status

Subject indexing assigned by CRD

Subject index terms

Adolescent; Adolescent Health; Anxiety; Anxiety Disorders; Depression; Disease Progression; Humans; Mental Disorders; Mental Health; Mental Health Recovery; Prognosis; Psychological Distress; Young Adult

Date of registration in PROSPERO

27 April 2021

Date of first submission

26 April 2021

Stage of review at time of this submission

Stage	Started	Completed
Preliminary searches	Yes	No
Piloting of the study selection process	No	No
Formal screening of search results against eligibility criteria	No	No
Data extraction	No	No
Risk of bias (quality) assessment	No	No
Data analysis	No	No

The record owner confirms that the information they have supplied for this submission is accurate and complete and they understand that deliberate provision of inaccurate information or omission of data may be construed as scientific misconduct.

The record owner confirms that they will update the status of the review when it is completed and will add publication details in due course.

Versions

27 April 2021
