# Supplementary material for: One-year recovery rates for young people with depression and/or anxiety not receiving treatment: a systematic review and meta-analysis
Source: BMJ Open. 2023 Jul 21;13(7):e072093. doi: 10.1136/bmjopen-2023-072093 (PMC10364186; doi:10.1136/bmjopen-2023-072093)
Supplement: Supplementary data [file bmjopen-2023-072093supp003.pdf]

Search strategy:

OVID (Embase, & Global Health)

1) Longitudinal OR Cohort OR `follow up` OR outcome* OR prognos* OR prospective OR retrospective AND

2) Adolescen* OR `young people` OR youth* OR teen* OR minor* OR juvenile* OR `young person*` OR `student*` OR `high school` OR `middle school` OR `secondary school` OR child* OR `young women` OR `young men` OR `boy*` OR `girl*` AND

3) `Mental distress` OR depress* OR anxiety OR anxious OR `psychological distress` OR `distress` OR `internal* disorder` OR `internal* behavio*` AND

4) `one year` OR `12 month*` OR `1 year`

5) 1 AND 2 AND 3 AND 4

MEDLINE (via PubMed):

(Longitudinal OR Cohort OR "follow up" OR outcome* OR prognos* OR prospective OR retrospective) AND (("Adolescent/psychology"[Mesh]) OR "young people" OR youth OR teen* OR minor OR juvenile* OR "young person" OR "student*" OR "high school" OR "middle school" OR "secondary school" OR child* OR "young women" OR "young men" OR boy OR girl*) AND ("Mental distress" OR ("Depression/diagnosis"[Mesh] OR "Depression/psychology"[Mesh] OR "Depression/rehabilitation"[Mesh] OR "Depression/therapy"[Mesh]) OR ("Anxiety/diagnosis"[Mesh] OR "Anxiety/psychology"[Mesh] OR "Anxiety/rehabilitation"[Mesh] OR "Anxiety/therapy"[Mesh]) OR ("Psychological Distress/diagnosis"[Mesh] OR "Psychological Distress/psychology"[Mesh] OR "Psychological Distress/rehabilitation"[Mesh] OR "Psychological Distress/therapy"[Mesh]) OR "distress" OR "internal* disorder" OR "internal* behavio*" OR ("Emotions/diagnosis"[Mesh] OR "Emotions/psychology"[Mesh] OR "Emotions/rehabilitation"[Mesh] OR "Emotions/therapy"[Mesh])) AND ("one year" OR "12 month*" OR "1 year")

MeSH Terms used:
"depression"
"anxiety"
"emotions"
"psychological distress"
"adolescent"

PsycINFO

(Longitudinal OR Cohort OR “follow up” OR outcome* OR prognos* OR prospective OR retrospective) AND (Adolescen* OR "young people" OR youth OR teen* OR minor OR juvenile* OR “young person” OR “student*” OR “high school” OR “middle school” OR “secondary school” OR child* OR “young women” OR “young men” OR boy* OR girl*) AND ("Mental distress" OR depress* OR anxiety OR anxious OR “psychological distress” OR “distress” OR “internal* disorder” OR internal* behavio*) AND ("one year" OR "12 month*" OR “1 year”)

Web of Science

(Longitudinal OR Cohort OR “follow up” OR outcome* OR prognos* OR prospective OR retrospective) AND (Adolescen* OR "young people" OR youth OR teen* OR minor OR juvenile* OR “young person” OR “student*” OR “high school” OR “middle school” OR “secondary school” OR child* OR “young women” OR “young men” OR boy* OR girl*) AND ("Mental distress" OR depress* OR anxiety OR anxious OR “psychological distress” OR “distress” OR “internal* disorder” OR internal* behavio*) AND ("one year" OR "12 month*" OR “1 year”)
