# Supplementary material for: One-year recovery rates for young people with depression and/or anxiety not receiving treatment: a systematic review and meta-analysis
Source: BMJ Open. 2023 Jul 21;13(7):e072093. doi: 10.1136/bmjopen-2023-072093 (PMC10364186; doi:10.1136/bmjopen-2023-072093)
Supplement: Supplementary data [file bmjopen-2023-072093supp005.pdf]

| Author, year | Country | Setting and Population | Study Aim | Age at baseline | Sample size at baseline | Measure of depressive and/or anxiety symptoms | Cut off scores | One year follow-up | Quality Assessment (NOS) |
|---|---|---|---|---|---|---|---|---|---|
| Fan et al., 2016 | China | High Schools in Dujiangyan, China; Population drawn from adolescents exposed to the 2008 Wenchuan earthquake in China. | This study explored the associations between posttraumatic stress disorder, depressive symptoms, and sleep disturbance after the Wenchuan earthquake | 13 to 17 years old | 455 participants | Chinese version of the Depression Self-Rating Scale for Children *(Self-report)* | Score of 15 has been used as the cut-off for screening depressive disorders | 292 participants recovered from depressive symptoms (64%) | 6 |
| Yang et al., 2008 | Taiwan | Middle Schools in Taiwan; population drawn from the students | This study evaluated the influence of personality traits and non-severe life events on depressive episodes in adolescents from six middle schools | 12 to 16 years old | 213 participants | Chinese version of the Centre for Epidemiologic Studies Depression *(Self-report)* | Wave 2 CES-D score becoming 28 or less by a decrease from Wave 1 CES-D score of >28 | 107 participants recovered (50%) | 6 |
| Goodyer et al., 2003 | U.K | Three secondary schools within Cambridge; population drawn from secondary school students who met the diagnostic criteria for major depressive disorder. | This study investigated whether hormones influence recovery in a community sample of adolescents with major depression | 12 to 16 years old | 30 participants (depressive symptoms) | Kiddie-SADS Schedule for Schizophrenia and Affective Disorders; Mood *(Clinician led scale)* Mood and Feelings Questionnaire *(Self-report)* | - | 19 participants recovered from depression (63%) | 7 |
| Leikanger, E & Larrson, B., 2012 | Norway | 18 schools across 17 municipalities in Sunnmøre county in western Norway; | This study explored the stability, incidences and changes in the DSM-IV related anxiety | 13 to 15 years old | 141 participants | The Screen for Child Anxiety Related Emotional Disorders (SCARED) Scale | Those who scored lower than 25 at baseline were defined as ‘‘low-moderate scorers’’, and those who scored 25 or | 67 participants had reduced symptoms of anxiety (48%) | 5 |

| | | | | | | | | | |
|---|---|---|---|---|---|---|---|---|---|
| | | population drawn from Norwegian adolescents | symptoms amongst Norwegian adolescents | | | *(Self-report)* | higher were defined as ''high scorers'' | | |
| Jinnin et al., 2016 | Japan | Hiroshima University in Japan; population drawn from first-year undergraduate students | This study looked at the progression of depressive symptoms and the potential risk for developing major depressive episodes in older adolescents | 18 to 19 years old | 172 participants | Japanese version of BDI-II<br><br>*(Self-report)* | Low-symptom group: BDI-II score ≤ 10<br><br>Middle-symptom group: BDI-II score = 11–17<br><br>High-symptom group: BDI-II score ≥ 18 | 81 participants had reduced symptoms of depression (47%) | 7 |

*Table 1. Characteristics of the included studies.*
