# Supplementary material for: One-year recovery rates for young people with depression and/or anxiety not receiving treatment: a systematic review and meta-analysis
Source: BMJ Open. 2023 Jul 21;13(7):e072093. doi: 10.1136/bmjopen-2023-072093 (PMC10364186; doi:10.1136/bmjopen-2023-072093)
Supplement: Supplementary data [file bmjopen-2023-072093supp004.pdf]

Risk of bias (quality) assessment

All the literature retrieved was critically appraised using common variables on an individual basis. Independent reviewers appraised methodological quality and risk of bias according to the Newcastle-Ottawa Scale (NOS) that has validity for use in cohort studies Wells et al. An eight-item scale with three quality parameters (i) selection, (ii) comparability and (iii) outcome. We rated the quality of the studies (good, fair, and poor) by allocating each domain with stars in this manner:

- A **Good** quality score was awarded 3 or 4 stars in selection, 1 or 2 in comparability and 2 or 3 stars in outcomes.
- A **Fair** quality score was awarded 2 stars in selection, 1 or 2 stars in comparability, and 2 or 3 stars in outcomes.
- A **Poor** quality score was awarded 0 or 1 star(s) in selection, 0 stars in comparability, and 0 or 1 star(s) in outcomes domain (Wells et al., 2000)

Newcastle-Ottawa Scale Quality Assessment of included studies (TABLE X)

**Author(s)**	**Selection**				**Comparability**		**Outcome**		
	**Representativeness of cohort**	**Selection of non-exposed**	**Ascertainment of exposure**	**Outcome of interest not present at start**	**Control for age and sex**	**For additional factors**	**Assessment of outcome**	**Length of follow-up**	**Adequacy of follow up**
Jinnin, R et al (2016)	*	-	*	*	**		-	*	*
Leikanger, E & Larsson, B (2012)	*	-	-	*	*		-	*	*
Goodyer, I.M & Herbert, J (2003)	*	-	*	*	**		-	*	*
Fan, F et al (2016)	*	-	*	*	*		-	*	*
Yang, H-J et al (2008)	*	-	-	*	**		-	*	*

Wells, G., Shea, B., O'Connel, D., Peterson, J, Welch, V., Loso, M., Tugwell, P. (2000). The Newcastle-ottawa scale (NOS) for assessing the quality of nonrandmoised studies in meta-analyses. Ottawa, ON, Canada: Ottawa Hospital Research Institute.
