# Supplementary material for: One-year recovery rates for young people with depression and/or anxiety not receiving treatment: a systematic review and meta-analysis
Source: BMJ Open. 2023 Jul 21;13(7):e072093. doi: 10.1136/bmjopen-2023-072093 (PMC10364186; doi:10.1136/bmjopen-2023-072093)
Supplement: Author's manuscript [file bmjopen-2023-072093.draft_revisions.pdf]

BMJ Open

BMJ Open is committed to open peer review. As part of this commitment we make the peer review history of every article we publish publicly available.

When an article is published we post the peer reviewers' comments and the authors' responses online. We also post the versions of the paper that were used during peer review. These are the versions that the peer review comments apply to.

The versions of the paper that follow are the versions that were submitted during the peer review process. They are not the versions of record or the final published versions. They should not be cited or distributed as the published version of this manuscript.

BMJ Open is an open access journal and the full, final, typeset and author-corrected version of record of the manuscript is available on our site with no access controls, subscription charges or pay-per-view fees (http://bmjopen.bmj.com).

If you have any questions on BMJ Open's open peer review process please email info.bmjopen@bmj.com

One-year recovery rates for young people with depression and/or anxiety not receiving treatment: A meta-analysis and systematic review

Journal:	*BMJ Open*
Manuscript ID	bmjopen-2023-072093
Article Type:	Original research
Date Submitted by the Author:	17-Feb-2023
Complete List of Authors:	Roach, Anna; Queen Mary University of London<br>Sureshkumar, Diliniya Stanislaus; Queen Mary University of London<br>Elliot, Kathryn; Queen Mary University of London<br>Hidalgo-Padilla, Liliana; Universidad Peruana Cayetano Heredia<br>van Loggerenberg, Francois; Youth Resilience Unit, Newham Centre for Mental Health ; Queen Mary University of London, Unit for Social and Community Psychiatry<br>Hounsell, Lauren; Queen Mary University of London<br>Jakaite, Zivile; Queen Mary University of London<br>Esnal, Fernando; Universidad de Buenos Aires<br>Donaghy, Jade; Queen Mary University of London<br>Bird, Victoria; Queen Mary University of London, Unit for Social and Community Psychiatry<br>Priebe, Stefan; Queen Mary University of London
Keywords:	MENTAL HEALTH, Child & adolescent psychiatry < PSYCHIATRY, Depression & mood disorders < PSYCHIATRY, Anxiety disorders < PSYCHIATRY

I, the Submitting Author has the right to grant and does grant on behalf of all authors of the Work (as defined in the below author licence), an exclusive licence and/or a non-exclusive licence for contributions from authors who are: i) UK Crown employees; ii) where BMJ has agreed a CC-BY licence shall apply, and/or iii) in accordance with the terms applicable for US Federal Government officers or employees acting as part of their official duties; on a worldwide, perpetual, irrevocable, royalty-free basis to BMJ Publishing Group Ltd ("BMJ") its licensees and where the relevant Journal is co-owned by BMJ to the co-owners of the Journal, to publish the Work in this journal and any other BMJ products and to exploit all rights, as set out in our licence.

The Submitting Author accepts and understands that any supply made under these terms is made by BMJ to the Submitting Author unless you are acting as an employee on behalf of your employer or a postgraduate student of an affiliated institution which is paying any applicable article publishing charge ("APC") for Open Access articles. Where the Submitting Author wishes to make the Work available on an Open Access basis (and intends to pay the relevant APC), the terms of reuse of such Open Access shall be governed by a Creative Commons licence – details of these licences and which Creative Commons licence will apply to this Work are set out in our licence referred to above.

Other than as permitted in any relevant BMJ Author's Self Archiving Policies, I confirm this Work has not been accepted for publication elsewhere, is not being considered for publication elsewhere and does not duplicate material already published. I confirm all authors consent to publication of this Work and authorise the granting of this licence.

For peer review only

One-year recovery rates for young people with depression and/or anxiety not receiving treatment: A meta-analysis and systematic review

Anna Roach, M.Sc[a], Diliniya Stanislaus Sureshkumar[a], Kathryn Elliot[a], Liliana Hidalgo-Padilla[b], Francois van Loggerenberg[c], Lauren Hounsell[a], Zivile Jakaite[a], Fernando Esnal[d], Jade Donaghy[a], Victoria Bird[a] and Stefan Priebe, M.S.(Psych), Ph.D[a]

[a] Unit for Social and Community Psychiatry (World Health Organization Collaborating Centre for Mental Health Services Development), Newham Centre for Mental Health, Queen Mary University of London, London, UK

[b] CRONICAS Center of Excellence in Chronic Diseases, Universidad Peruana Cayetano Heredia, Lima, Peru

[c] Youth Resilience Unit (YRU), Newham Centre for Mental Health, Queen Mary University of London, London, UK

[d] Universidad de Buenos Aires, Buenos Aries, Argentina

Address correspondence: Anna Roach, Unit of Social and Community Psychology, Newham Centre for Mental Health, Queen Mary University of London; Email address: anna.roach.21@ucl.ac.uk

Word count: 3161

For peer review only - http://bmjopen.bmj.com/site/about/guidelines.xhtml

ABSTRACT

Objective: To systematically review one-year recovery rates for young people experiencing depression and/or anxiety who are not receiving any specific mental health treatment.

Method: Five databases were searched for articles. Eligible articles were peer reviewed, published in English and had baseline and one-year follow up depression and/or anxiety outcomes for young people aged 10-24 years without specific treatment.

Results: Of the 17,250 references screened for inclusion, five articles with 1,011 participants in total were included. Studies reported a one-year recovery rate of between 47-64%. In the meta-analysis, the overall pooled proportion of recovered young people is 0.54 (0.45 – 0.63).

Conclusion: The findings suggest that after one-year about 54% of young people with symptoms of anxiety and/or depression recover without any specific mental health treatment. Future research should identify individual characteristics predicting recovery and explore resources and activities which may help young people recover from depression and/or anxiety.

PROSPERO registration number: CRD42021251556

Data availability statement: Data sharing not applicable as no datasets generated and/or analysed for this study. All data relevant to the study are included in the article or uploaded as online supplemental information. All data underlying the results are available as part of the article and no additional source data are required.

Key words: Depression, anxiety, recovery, one-year outcomes, young people

STRENGTHS AND LIMITATION OF THIS STUDY

- Although there is increasing amounts of research surrounding which young people get ill, there is very limited understanding of what happens when they do. This is the first systematic review and meta-analysis to explore recovery rates after one year of young people with depression and/or generalised anxiety
- Symptom reduction across depression and generalised anxiety was shown in similar proportions across the different studies
- Evidence base with data of a total of 1,011 participants is limited, and the small number of studies meant moderator analyses could not be conducted
- No available information about how long symptoms had been present for at baseline meaning participants with very short episodes of depression and/or anxiety were likely missed

For peer review only - http://bmjopen.bmj.com/site/about/guidelines.xhtml

INTRODUCTION

Adolescence and young adulthood are important developmental periods of social, behavioural, and psychological change where there is a transition from the dependence of childhood to adulthood independence [1]. However, these periods are also associated with increased risk of experiencing mental distress with most functional mental disorders beginning before the age of 25 years [2]. Research suggests that the majority of young people are likely to experience mental distress at some point of time [3], yet the spontaneous prognosis of such distress remains uncertain. An understanding of the prognosis without treatment may inform appropriate responses on individual and public health levels [4].

Improving mental health among young people has been identified by the World Health Organisation (WHO) as a key priority to promote social and economic development [5]. Experiencing depression and anxiety in early life is associated with poor outcomes, such as high levels of distress and disability, future physical and psychiatric morbidity, and educational and social impairment [6-8]. Strengthening the resilience of young people is suggested as key to promoting long term mental health outcomes [9].

Although some research estimates that a proportion of those who experience depressive episodes in adolescence will go on to experience at least one recurrent episode in adulthood [10], to date there has been no systematic exploration of the one-year recovery outcomes of adolescent depression and/or anxiety.

Against this background, we conducted a systematic review and meta-analysis of recovery rates of young people with depression and/or generalised anxiety who did not receive any specific mental health treatment. For an inclusive approach, we used a wide age range including adolescence and young adulthood, i.e. 10-24 years, following the WHO definition of young people [11]. The one-year time frame appears relevant for considering whether young people with symptoms of depression and/or anxiety should be referred to mental health services and has the pragmatic advantage that studies tend to report outcomes for one-year rather than for other timeframes.

METHODS

We conducted a systematic review and meta-analysis to estimate the proportion of young people who recover from depression and anxiety in a one-year period. Methodology and reporting for this systematic review are consistent with PRISMA guidelines [12]. A PRISMA checklist is provided in *Supplementary Material 1*. A protocol for this review was developed a priori and registered on PROSPERO (CRD42021251556) and is available in *Supplementary Material 2*.

Search strategy

A comprehensive literature search was conducted in five electronic databases: MEDLINE, Embase, PsycINFO, Web of Science, and Global Health for articles published from 1980 through to August 2022 The search strategy was adapted for the different databases and search terms, included the study design (longitudinal, cohort, prospective, etc), terms relating to young people (adolescents, youth, school, etc), terms encompassing mental distress (depression, anxiety, internalising disorder, etc) and a one year or 12 month follow up. An example search strategy and the MeSH headings used are available in *Supplementary Material 3.* The reference and citation lists were also scanned of eligible articles to supplement our database searches.

Eligibility criteria

For peer review only - http://bmjopen.bmj.com/site/about/guidelines.xhtml

Articles were eligible if they included

- Participants defined by WHO as “young people” (aged 10-24) at baseline, which includes adolescents (aged 10-19) and youth (aged 15-24).
- A validated self-report or observer-rated measure of depression and/or generalised anxiety or a diagnosis of depression and/or generalised anxiety as defined by the ICD-10 or equivalent.
- A prospective design including one year follow-up depression and/or anxiety outcomes.

Only studies reporting individual recovery rates of anxiety and/or depression were included, studies reporting group recovery rates were not included unless the whole sample had symptoms with anxiety and/or depression at baseline. Furthermore, included articles had to be published in English in peer-reviewed journals with longitudinal cohort research study designs (with one-year follow-up data available).

We excluded all studies where young people were receiving pharmacological or psychological treatment or other specific interventions for their mental health. Grey literature was also not included.

Risk of bias

The methodological quality of eligible articles were assessed using the Newcastle-Ottawa Scale (NOS) [13] a quality assessment tool used for non RCT studies including case-control and cohort studies. The NOS uses a star rating system; stars are assigned for eight items grouped into three categories assessing selection of study groups, comparability, and outcomes.

Patient and Public Involvement

No patients involved.

RESULTS

A search in five electronic databases identified 17,250 articles of which 16,200 were excluded based on the eligibility criteria *(Fig. 1, PRISMA Diagram)*. In total, five studies were included in our analysis reporting recovery data of 1,011 participants. Many studies were excluded as they showed a general trend in recovery rather than reporting individual recovery rate. All studies included were longitudinal studies that examined the one-year outcomes of depressive and/or anxiety symptoms in young people. These papers are summarised in *Table 1*.

A large team of independent reviewers (AR, DSS, LH, FvL, ZJ, LH, FE, JD) screened titles and abstracts to ascertain potential eligibility. Those appearing relevant underwent full-text review, of which 20% were double screened (KE). The disagreement rate was 3%, and the conflicts were resolved by an independent reviewer (FvL).

Data Extraction

Three reviewers (AR, DSS and KE) independently extracted data from eligible articles into a computerised extraction form developed before review (available on request). Data extracted included study objective, country/setting of research, sample size, baseline outcome measure, validated measure of depressive and/or anxiety symptoms (including if self-report/clinician led), age at baseline, follow up duration, follow up outcome, method of analysis, and covariates. Several papers did not provide the required information for this review; thus, the authors of each study were contacted to gain the necessary details. There were four authors that did not reply and

For peer review only - http://bmjopen.bmj.com/site/about/guidelines.xhtml

therefore those papers were excluded from the analysis. Study characteristics are summarised in *Table 1*.

Figure 1: PRISMA flowchart of paper selection process

Data Analysis

A meta-analysis was conducted within STATA to calculate the proportion of individuals classified as recovered. We used Wilson's method to calculate 95% confidence intervals for proportion estimates. The approach produces asymmetric confidence intervals in studies with low proportion rates. Heterogeneity among studies was estimated based on Cochran Q and reported using I^2 (and 95% confidence interval of the I^2). $I^2 > 75\%$ is considered indicative of high heterogeneity. Given the heterogeneity within the papers, we used a random effects meta-analysis applying the metan code within STATA Version 15 (StataCorp LP, College Station, Texas).

As it is common for meta-analyses of observational or cohort data to report high levels of heterogeneity, prediction intervals were also calculated. The prediction interval includes the plausible range of estimates we would expect the effect of a new study to be within. They are wider than confidence intervals as they include the random variation of individual measurement as well as the uncertainty of estimating the population effect size. To calculate the prediction intervals, the "meta set" command was first used to declare the data as meta data, this was followed by the "meta summarize" and "predinterval" commands.

Study Characteristics

A total of 1,011 participants were included in this systematic review. The sample size ranged from 30 to 455 with the age at baseline varying from 12–19. Of the five papers in this review four were from high-income countries [14-17] and one from a middle-income country [18]. The majority of the studies reported the one year outcome of depression [14-18] with only one study observing the outcomes of anxiety in young people [16]. All young people met either a threshold for elevated symptoms or diagnosis of depression and/or anxiety at baseline, as defined by the authors of the respective studies. All participants in these studies were recruited from education settings [14-18].The majority of the measures used were self-reporting scales, except for the Kiddie-SADS measure which is clinician led.

Risk of bias

The quality appraisal of the five studies is presented in *Table 1*, with three of the studies assessed to be of good quality scoring (i.e. scoring 3 or 4 stars in selection, 1 or 2 in comparability and 2 or 3 stars in outcomes) and only two studies deemed to be of fair reporting quality (i.e. scoring 2 stars in selection, 1 or 2 stars in comparability, and 2 or 3 stars in outcomes). All studies were deemed representative of the target population and controlled for various confounding variables. The five studies all stated a one-year follow up period with little to no follow-up loss. Three studies administered structured interviews to ascertain the exposure; however, none of the studies used a formal assessment to determine the outcome as they relied upon the use of self-reported measures *(see Supplementary material 4 for full quality appraisal)*.

For peer review only - http://bmjopen.bmj.com/site/about/guidelines.xhtml

Author, year	Country	Setting and Population	Study Aim	Age at baseline	Sample size at baseline	Measure of depressive and/or anxiety symptoms	Cut off scores	One year follow-up	Quality Assessment (NOS)
Fan et al., 2016	China	High Schools in Dujiangyan, China; Population drawn from adolescents exposed to the 2008 Wenchuan earthquake in China.	This study explored the associations between posttraumatic stress disorder, depressive symptoms, and sleep disturbance after the Wenchuan earthquake	13 to 17 years old	455 participants	Chinese version of the Depression Self-Rating Scale for Children *(Self-report)*	Score of 15 has been used as the cut-off for screening depressive disorders	292 participants recovered from depressive symptoms (64%)	6
Yang et al., 2008	Taiwan	Middle Schools in Taiwan; population drawn from the students	This study evaluated the influence of personality traits and non-severe life events on depressive episodes in adolescents from six middle schools	12 to 16 years old	213 participants	Chinese version of the Centre for Epidemiologic Studies Depression *(Self-report)*	Wave 2 CES-D score becoming 28 or less by a decrease from Wave 1 CES-D score of >28	107 participants recovered (50%)	6
Goodyer et al., 2003	U.K	Three secondary schools within Cambridge; population drawn from secondary school students who met the diagnostic criteria for major depressive disorder.	This study investigated whether hormones influence recovery in a community sample of adolescents with major depression	12 to 16 years old	30 participants (depressive symptoms)	Kiddie-SADS Schedule for Schizophrenia and Affective Disorders; Mood *(Clinician led scale)* Mood and Feelings Questionnaire *(Self-report)*	-	19 participants recovered from depression (63%)	7

For peer review only - http://bmjopen.bmj.com/site/about/guidelines.xhtml

Leikanger, E & Larrson, B., 2012	Norway	18 schools across 17 municipalities in Sunnmøre county in western Norway; population drawn from Norwegian adolescents	This study explored the stability, incidences and changes in the DSM-IV related anxiety symptoms amongst Norwegian adolescents	13 to 15 years old	141 participants	The Screen for Child Anxiety Related Emotional Disorders (SCARED) Scale<br><br>*(Self-report)*	Those who scored lower than 25 at baseline were defined as ''low-moderate scorers'', and those who scored 25 or higher were defined as ''high scorers''	67 participants had reduced symptoms of anxiety (48%)	5
Jinnin et al., 2016	Japan	Hiroshima University in Japan; population drawn from first-year undergraduate students	This study looked at the progression of depressive symptoms and the potential risk for developing major depressive episodes in older adolescents	18 to 19 years old	172 participants	Japanese version of BDI-II<br><br>*(Self-report)*	Low-symptom group: BDI-II score ≤ 10<br><br>Middle-symptom group: BDI-II score = 11–17<br><br>High-symptom group: BDI-II score ≥ 18	81 participants had reduced symptoms of depression (47%)	7

Table 1. Characteristics of the included studies.

For peer review only - http://bmjopen.bmj.com/site/about/guidelines.xhtml

For peer review only

One-year recovery

Five studies provided data for the meta-analysis *(see Figure 2)*. The overall pooled proportion of young people to recover (defined as symptom reduction or remission) is 0.54 (0.45 – 0.63), which indicates roughly half (54%) of young people recover without any specific mental health treatment or intervention within a year. Although there is substantial heterogeneity across the eligible studies (I^2=84%), to account for the high heterogeneity evident in meta-analyses of observation data, prediction intervals were also calculated (90% PI 0.340, 0.738), to estimate an interval range in which a future observation will fall.

Figure 2: Forest plot showing pooled proportions and 95% confidence intervals (CIs) for recovery rate and symptoms reduction of anxiety and/or depression for young people, after 1 year.

All five studies reported a recovery rate ranging from 47% to 64%. There was only one study that looked at the recovery of symptoms of anxiety and they found that 48% of young people (95% CI 0.39-0.56) had gone from scoring high on the SCARED scale to low-moderate after a year [16].

Gender differences for recovery of young people with depression were reported in only one study [16], who reported that 67 adolescents (47.5 % of the high scorers at baseline: 47.8 % of girls, and 46.4 % of boys) had high scores at baseline but low to moderate scores at 12-month follow up.

DISCUSSION

The findings of this systematic review and meta-analysis indicate that 54% of young people with symptoms of depression and/or anxiety tend to achieve recovery, defined as symptom reduction below a threshold within a year, when they do not receive specific mental health treatment. The included studies show relatively consistent results with limited variation. Based on the available evidence, the most likely spontaneous prognosis for young people with symptoms of depression and/or anxiety is that about half of them will have recovered after a year. Such process can be understood as a sign of resilience with young people bouncing back from their experience of distress [19].

There is no systematic review in adults that offers a direct comparison, however literature suggests that minor depression is likely to persist and poses an elevated risk of worsening over one year in 60% of older adults [20].

Strengths and limitations

This is the first systematic review and meta-analysis to explore one-year recovery rates of young people with depression and/or anxiety. Symptom reduction across depression and anxiety was shown in similar proportions across the different studies, with markedly small margins which suggest relatively robust findings. Pooled analyses were therefore representative of papers and one study with a larger sample size was not skewing the results. Finally, most articles included were of moderate to high methodologic quality and used validated measures with strong psychometric properties. Previous research suggests adolescents are capable of providing valid self-reports of depressive symptoms [21], strengthening the reliability of our findings.

This review does however have several limitations. Firstly, we have no systematic information about how long symptoms had been present for at baseline. Studies started with cross-sectional identification of participants who met the symptom levels defined as inclusion criteria at the time of recruitment to the cohort study. These studies were likely to miss participants with very short episodes of depression and/or anxiety and recruit participants whose symptoms had already been

For peer review only - http://bmjopen.bmj.com/site/about/guidelines.xhtml

there for longer. Thus, the spontaneous prognosis of young people experiencing their first symptoms of depression and/or anxiety is likely to be more favourable than the 54% in this review. Secondly, given the world-wide interest in the mental health of young people, the evidence base with data of a total of 1,011 participants is limited, and there was only one study exploring recovery rates of anxiety. It was not possible to access data from the control groups or “treatment as usual” groups from randomised controlled trials, and due to the small number of included studies, moderation analysis on patient factors such as age and gender could not be conducted. Thirdly, we focused on only two time points, i.e. baseline and one-year follow-up, and did not consider what happened in between the two time points or after the follow-up. For instance, participants who had symptoms after a year might have been symptom-free in between, and participants we regarded as recovered may have relapsed after the follow-up. The findings do therefore not imply that 54% will remain recovered in the future. Finally, the studies included in this review are highly heterogeneous. Although this suggests that similar recovery rates can be found in very different contexts, it may also challenge whether it is appropriate to derive an overall estimate from that set of studies. The data was also not sufficient to distinguish between narrower age bands within the wide group of young people.

Implications

Although there is evidence that adolescents with depression go on to experience episodes of depression in later life [22] our review suggests that significant symptom reduction occurs after one year in at least half of young people who are affected. Three to nine percent of teenagers meet the criteria for depression at any one time [23], and the question arises as to whether they should routinely be considered for specialised treatments or whether one should wait with such decisions for year by which time about 54% are likely to have recovered without treatment.

A recent systematic review found that although psychotherapies for depression in young people can be effective, more than 60% of those receiving therapy do not respond [24]. Similarly, antidepressants have been found to have only a small therapeutic effect in this group [25]. The decision as to whether specific treatments should be considered is further complicated by the absence of evidence-based predictor variables for a) who would or would not recover without treatment, and b) who would or would not benefit from specific treatments. School-based and community programs are gaining traction, especially following the COVID-19 pandemic [26] but further studies are needed to demonstrate long term outcomes and successful implementation into routine practice.

It is important to analyse and further understand why more than half of young people recover, while other young people continue to report symptoms of depression and anxiety. Further research should identify personal characteristics predicting recovery. Since the predictive value of common socio-demographic characteristics is likely to be limited, such research may have to consider individual experiences, attitudes, family cohesion and community links.

The main task for future research however may be to explore what personal, family and community resources young people can find and utilise that help them recover from depression and anxiety. Such research may lead to social and public health interventions that can make a difference to large groups of affected young people and substantially improve the recovery rate of 54% found in this review.

For peer review only - http://bmjopen.bmj.com/site/about/guidelines.xhtml

Given the relatively small variability of recovery rates in this review, future intervention studies may use the 54% finding of this study as a yardstick to evaluate outcomes, when recruiting and assessing control groups is impractical or impossible.

CONCLUSION

In conclusion, although it is encouraging to find that half of young people tend to recover without treatment, if almost 50% still have symptoms after one year it does raise concerns. This is especially so as previous research shows only a small effect of both pharmacological and psychological interventions, although such treatments through mental health professionals generate significant costs. This calls for further development and evaluation of treatments in mental health care, but also interventions delivered within families, schools, and communities that prevent mental distress in young people as far as possible and help those experiencing symptoms of depression or anxiety to recover.

Contributor statement: AR and SP came up with the research question. AR, DS and LH-P led data extraction with KE, FvL, LH, ZJ, FE and JD assisting with title and abstract screening and then full text screening. AR, DS, KE and VB analysed the included papers. AR and DS drafted the manuscript and all provided feedback on the paper before submission.

Competing interests: The authors declared no potential conflicts of interest with respect to the research, authorship, and/or the publication of this article.

Funding: This research did not receive any specific grant from funding agencies in the public, commercial, or not-for-profit sectors.

Data sharing statement: Data sharing not applicable as no datasets generated and/or analysed for this study. All data relevant to the study are included in the article or uploaded as online supplemental information. All data underlying the results are available as part of the article and no additional source data are required.

Ethics approval statement: Ethical approval was not required for this systematic review and meta-analysis.

For peer review only

References

1. Priebe S, Fung C, Brusco LI, et al. Which resources help young people to prevent and overcome mental distress in deprived urban areas in Latin America? A protocol for a prospective cohort study. BMJ open 2021;11(9):e052339.

2. Solmi M, Radua J, Olivola M, et al. Age at onset of mental disorders worldwide: large-scale meta-analysis of 192 epidemiological studies. Molecular psychiatry 2022;27(1):281-95.

3. Kessler RC, Amminger GP, Aguilar-Gaxiola S, Alonso J, Lee S, Ustun TB. Age of onset of mental disorders: a review of recent literature. Current opinion in psychiatry 2007;20(4):359.

4. Patton GC, Coffey C, Romaniuk H, et al. The prognosis of common mental disorders in adolescents: a 14-year prospective cohort study. The Lancet 2014;383(9926):1404-11.

5. Varese F, Smeets F, Drukker M, et al. Childhood adversities increase the risk of psychosis: a meta-analysis of patient-control, prospective-and cross-sectional cohort studies. Schizophrenia bulletin 2012;38(4):661-71.

6. Fogel J, Eaton W, Ford D. Minor depression as a predictor of the first onset of major depressive disorder over a 15-year follow-up. Acta Psychiatrica Scandinavica 2006;113(1):36-43.

7. Goodman E, Huang B. Socioeconomic status, depressive symptoms, and adolescent substance use. Archives of pediatrics & adolescent medicine 2002;156(5):448-53.

8. Merikangas KR, He J-p, Burstein M, et al. Lifetime prevalence of mental disorders in US adolescents: results from the National Comorbidity Survey Replication–Adolescent Supplement (NCS-A). Journal of the American Academy of Child & Adolescent Psychiatry 2010;49(10):980-89.

9. Anderson K, Priebe S. Concepts of resilience in adolescent mental health research. Journal of Adolescent Health 2021;69(5):689-95.

10. Johnson D, Dupuis G, Piche J, Clayborne Z, Colman I. Adult mental health outcomes of adolescent depression: A systematic review. Depression and anxiety 2018;35(8):700-16.

11. WHO. Adolescent Health. Secondary Adolescent Health 2021. https://www.who.int/southeastasia/health-topics/adolescent-health.

12. Liberati A, Altman DG, Tetzlaff J, et al. The PRISMA statement for reporting systematic reviews and meta-analyses of studies that evaluate health care interventions: explanation and elaboration. Journal of clinical epidemiology 2009;62(10):e1-e34.

13. Wells GA, Shea B, O'Connell D, et al. The Newcastle-Ottawa Scale (NOS) for assessing the quality of nonrandomised studies in meta-analyses: Oxford, 2000.

14. Fan F, Zhou Y, Liu X. Sleep disturbance predicts posttraumatic stress disorder and depressive symptoms: a cohort study of Chinese adolescents. The Journal of clinical psychiatry 2017;78(7):19701.

15. Goodyer I, Herbert J, Tamplin A. Psychoendocrine antecedents of persistent first-episode major depression in adolescents: a community-based longitudinal enquiry. Psychological medicine 2003;33(4):601-10.

For peer review only - http://bmjopen.bmj.com/site/about/guidelines.xhtml

16. Leikanger E, Larsson B. One-year stability, change and incidence in anxiety symptoms among early adolescents in the general population. European Child & Adolescent Psychiatry 2012;21(9):493-501.

17. Jinnin R, Okamoto Y, Takagaki K, et al. Detailed course of depressive symptoms and risk for developing depression in late adolescents with subthreshold depression: a cohort study. Neuropsychiatric Disease and Treatment 2017;13:25.

18. Yang H-J, Chiu Y-J, Soong W-T, Chen WJ. The roles of personality traits and negative life events on the episodes of depressive symptoms in nonreferred adolescents: a 1-year follow-up study. Journal of Adolescent Health 2008;42(4):378-85.

19. Windle G. What is resilience? A review and concept analysis. Reviews in clinical gerontology 2011;21(2):152-69.

20. Lyness JM, Chapman BP, McGriff J, Drayer R, Duberstein PR. One-year outcomes of minor and subsyndromal depression in older primary care patients. International Psychogeriatrics 2009;21(1):60-68.

21. Moretti M, Fine S, Haley G, Marriage K. Childhood and adolescent depression: Child-report versus parent-report information. Journal of the American Academy of Child Psychiatry 1985;24(3):298-302.

22. Clayborne ZM, Varin M, Colman I. Systematic review and meta-analysis: adolescent depression and long-term psychosocial outcomes. Journal of the American Academy of Child & Adolescent Psychiatry 2019;58(1):72-79.

23. Bansal V, Goyal S, Srivastava K. Study of prevalence of depression in adolescent students of a public school. Industrial psychiatry journal 2009;18(1):43.

24. Cuijpers P, Karyotaki E, Ciharova M, et al. The effects of psychological treatments of depression in children and adolescents on response, reliable change, and deterioration: a systematic review and meta-analysis. European Child & Adolescent Psychiatry 2021:1-16.

25. Vitiello B, Ordóñez AE. Pharmacological treatment of children and adolescents with depression. Expert opinion on pharmacotherapy 2016;17(17):2273-79.

26. Hamoda HM, Chiumento A, Alonge O, et al. Addressing the consequences of the COVID-19 lockdown for children's mental health: Investing in school mental health programs. Psychiatric services 2021;72(6):729-31.

For peer review only

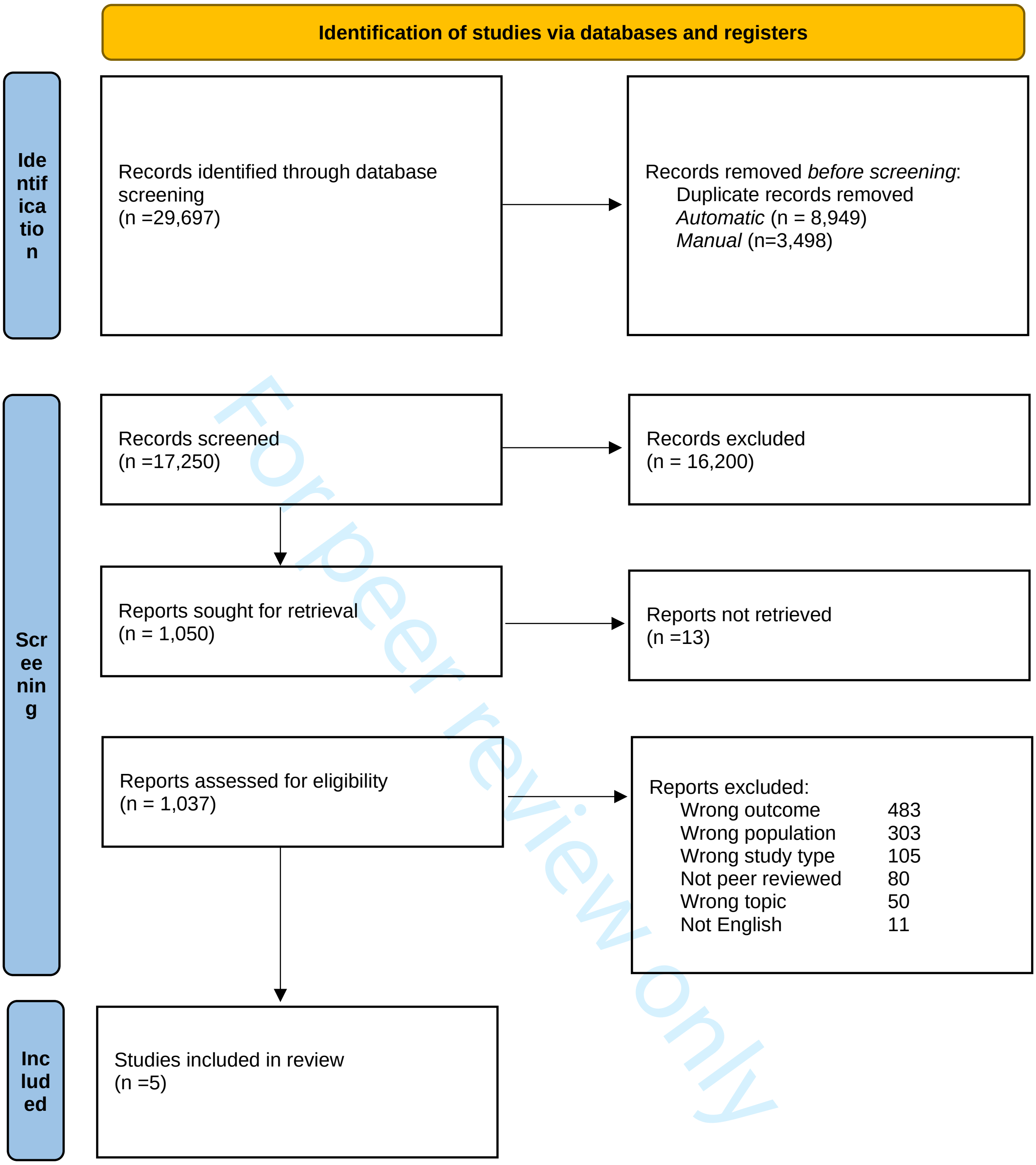

Figure 1 – PRISMA flowchart of paper selection process

For peer review only

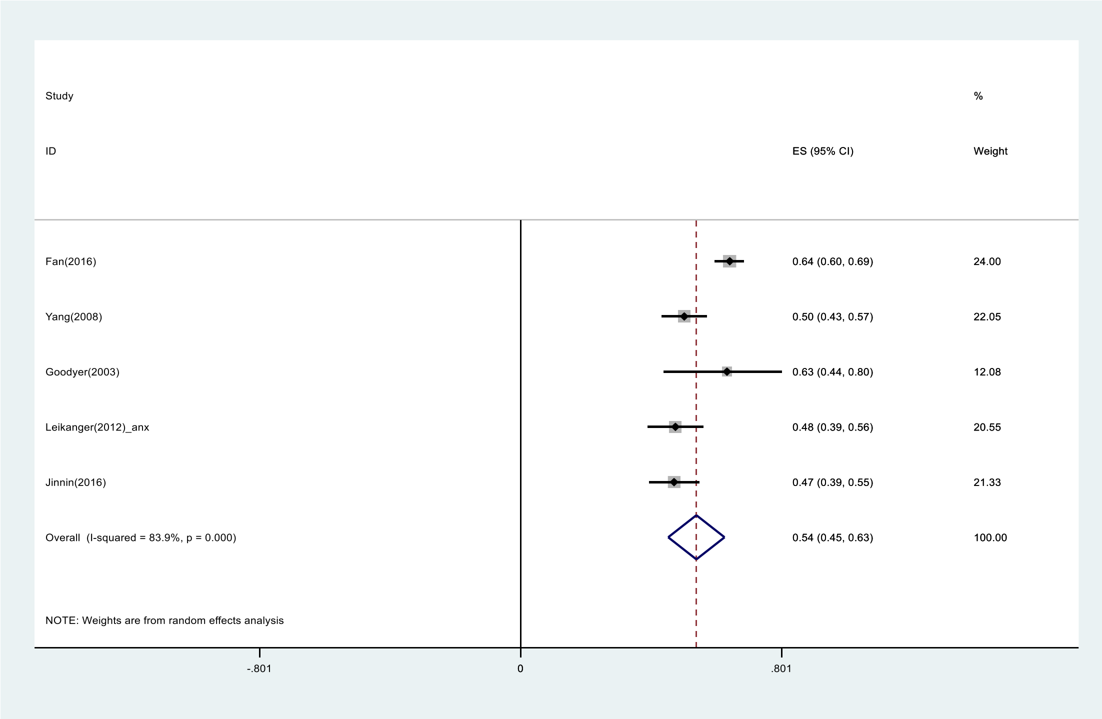

Figure 2 - Forest plot showing pooled proportions and 95% confidence intervals (CIs) for recovery rate and symptoms reduction of anxiety and/or depression for young people, after 1 year.

PRISMA 2020 Checklist

Section and Topic	Item #	Checklist item	Location where item is reported
**TITLE**			
Title	1	Identify the report as a systematic review.	P1
**ABSTRACT**			
Abstract	2	See the PRISMA 2020 for Abstracts checklist.	P1
**INTRODUCTION**			
Rationale	3	Describe the rationale for the review in the context of existing knowledge.	P2/3
Objectives	4	Provide an explicit statement of the objective(s) or question(s) the review addresses.	P2/3
**METHODS**			
Eligibility criteria	5	Specify the inclusion and exclusion criteria for the review and how studies were grouped for the syntheses.	P3/4
Information sources	6	Specify all databases, registers, websites, organisations, reference lists and other sources searched or consulted to identify studies. Specify the date when each source was last searched or consulted.	P3
Search strategy	7	Present the full search strategies for all databases, registers and websites, including any filters and limits used.	Suppl2
Selection process	8	Specify the methods used to decide whether a study met the inclusion criteria of the review, including how many reviewers screened each record and each report retrieved, whether they worked independently, and if applicable, details of automation tools used in the process.	P3
Data collection process	9	Specify the methods used to collect data from reports, including how many reviewers collected data from each report, whether they worked independently, any processes for obtaining or confirming data from study investigators, and if applicable, details of automation tools used in the process.	P3/4
Data items	10a	List and define all outcomes for which data were sought. Specify whether all results that were compatible with each outcome domain in each study were sought (e.g. for all measures, time points, analyses), and if not, the methods used to decide which results to collect.	P5
	10b	List and define all other variables for which data were sought (e.g. participant and intervention characteristics, funding sources). Describe any assumptions made about any missing or unclear information.	NA
Study risk of bias assessment	11	Specify the methods used to assess risk of bias in the included studies, including details of the tool(s) used, how many reviewers assessed each study and whether they worked independently, and if applicable, details of automation tools used in the process.	P4, P9 Suppl3
Effect measures	12	Specify for each outcome the effect measure(s) (e.g. risk ratio, mean difference) used in the synthesis or presentation of results.	P5 & Table 1
Synthesis methods	13a	Describe the processes used to decide which studies were eligible for each synthesis (e.g. tabulating the study intervention characteristics and comparing against the planned groups for each synthesis (item #5)).	P5 & Table 1
	13b	Describe any methods required to prepare the data for presentation or synthesis, such as handling of missing summary statistics, or data conversions.	P7
	13c	Describe any methods used to tabulate or visually display results of individual studies and syntheses.	P7 & Table 1
	13d	Describe any methods used to synthesize results and provide a rationale for the choice(s). If meta-analysis was performed, describe the model(s), method(s) to identify the presence and extent of statistical heterogeneity, and software package(s) used.	P7
	13e	Describe any methods used to explore possible causes of heterogeneity among study results (e.g. subgroup analysis, meta-regression).	P7
	13f	Describe any sensitivity analyses conducted to assess robustness of the synthesized results.	NA
Reporting bias assessment	14	Describe any methods used to assess risk of bias due to missing results in a synthesis (arising from reporting biases).	NA

For peer review only - http://bmjopen.bmj.com/site/about/guidelines.xhtml

PRISMA 2020 Checklist

Section and Topic	Item #	Checklist item	Location where item is reported
Certainty assessment	15	Describe any methods used to assess certainty (or confidence) in the body of evidence for an outcome.	P7
**RESULTS**			
Study selection	16a	Describe the results of the search and selection process, from the number of records identified in the search to the number of studies included in the review, ideally using a flow diagram.	Figure 1
	16b	Cite studies that might appear to meet the inclusion criteria, but which were excluded, and explain why they were excluded.	P4
Study characteristics	17	Cite each included study and present its characteristics.	Table 1
Risk of bias in studies	18	Present assessments of risk of bias for each included study.	Table 1
Results of individual studies	19	For all outcomes, present, for each study: (a) summary statistics for each group (where appropriate) and (b) an effect estimate and its precision (e.g. confidence/credible interval), ideally using structured tables or plots.	Table 1
Results of syntheses	20a	For each synthesis, briefly summarise the characteristics and risk of bias among contributing studies.	Table 1
	20b	Present results of all statistical syntheses conducted. If meta-analysis was done, present for each the summary estimate and its precision (e.g. confidence/credible interval) and measures of statistical heterogeneity. If comparing groups, describe the direction of the effect.	Figure 2
	20c	Present results of all investigations of possible causes of heterogeneity among study results.	NA
	20d	Present results of all sensitivity analyses conducted to assess the robustness of the synthesized results.	NA
Reporting biases	21	Present assessments of risk of bias due to missing results (arising from reporting biases) for each synthesis assessed.	NA
Certainty of evidence	22	Present assessments of certainty (or confidence) in the body of evidence for each outcome assessed.	NA
**DISCUSSION**			
Discussion	23a	Provide a general interpretation of the results in the context of other evidence.	P11-13
	23b	Discuss any limitations of the evidence included in the review.	P11
	23c	Discuss any limitations of the review processes used.	P11
	23d	Discuss implications of the results for practice, policy, and future research.	P12-13
**OTHER INFORMATION**			
Registration and protocol	24a	Provide registration information for the review, including register name and registration number, or state that the review was not registered.	P1&3
	24b	Indicate where the review protocol can be accessed, or state that a protocol was not prepared.	P1&3
	24c	Describe and explain any amendments to information provided at registration or in the protocol.	NA
Support	25	Describe sources of financial or non-financial support for the review, and the role of the funders or sponsors in the review.	NA
Competing interests	26	Declare any competing interests of review authors.	NA
Availability of data, code and other materials	27	Report which of the following are publicly available and where they can be found: template data collection forms; data extracted from included studies; data used for all analyses; analytic code; any other materials used in the review.	NA

For peer review only

PRISMA 2020 Checklist

From: Page MJ, McKenzie JE, Bossuyt PM, Boutron I, Hoffmann TC, Mulrow CD, et al. The PRISMA 2020 statement: an updated guideline for reporting systematic reviews. BMJ 2021;372:n71. doi: 10.1136/bmj.n71

For more information, visit: http://www.prisma-statement.org/

For peer review only

One year outcomes for young people with depression and/or anxiety: a systematic review

Citation

Anna Roach, Diliniya Sureshkumar, Stefan Priebe. One year outcomes for young people with depression and/or anxiety: a systematic review. PROSPERO 2021 CRD42021251556 Available from: https://www.crd.york.ac.uk/prospero/display_record.php?ID=CRD42021251556

Review question

What are the one year outcomes for young people with depression and/or anxiety?

Searches

Five databases will be searched: MEDLINE via PubMed, PsycINFO, Web of Science, Embase, Global Health.

MeSH terms to be used: "depression" "anxiety" "emotions" "psychological distress" "adolescent".

Inclusion:

• Longitudinal cohort research studies (with one year follow-up data);

• Texts must include primary data on anxiety and/or depression.

Exclusion:

• Studies of a specific clinical intervention/treatment;

• Grey literature (due to lack of peer-review).

The search will be re-run prior to submission.

Types of study to be included

Inclusion:

• Longitudinal cohort research studies (with one year follow-up data);

• Texts must include primary data on anxiety and/or depression.

Studies from clinical and non-clinical contexts will be included.

Research from any country will be included.

Exclusion:

• Studies of a specific clinical intervention/treatment;

For peer review only

• Grey literature (due to lack of peer-review).

Condition or domain being studied

Depression and/or anxiety in young people.

Participants/population

Inclusion:

- Participants defined by WHO as “young people” (aged 10-24) at baseline. This includes adolescents (aged 10-19) and youth (aged 15-24);

- Young people with baseline symptom levels above a specified clinical cut off point and/or a diagnosis of depression and/or anxiety as defined by the ICD-10 or equivalent. This will be indicated by a validated self-report or observer-rated questionnaire or interview.

Exclusion:

- None.

Intervention(s), exposure(s)

The focus of this review is assessing one year outcomes for young people experiencing depression and/or anxiety.

Comparator(s)/control

Not applicable.

Context

Main outcome(s)

Main outcome: recovery rate.

Secondary outcome: symptom level change.

Additional outcome(s)

Measures of quality of life and school and work performance, and social functioning will be included in our analysis of secondary outcomes.

Data extraction (selection and coding)

A minimum of two researchers will be involved in the screening, appraisal, and data extraction. Titles and abstracts will initially be screened by one author and duplicates removed. A second review author will independently screen 25% of these to check for consistency.

The full texts of potentially eligible studies will then be retrieved and assessed for eligibility by one author, and a second researcher will screen a random subset of 25% of potentially eligible studies. Any disagreements will be resolved by discussion with a third author.

MS Excel will be used to create a data extraction table. Extracted information will include study objective,

For peer review only

country/setting of research, sample size, baseline outcome measure, validated measure of depressive and/or anxious symptoms (including if self-report/clinician led), age at baseline, follow up duration, follow up outcome, method of analysis, covariates and notes.

Risk of bias (quality) assessment

Quality assessment will be conducted by a minimum of two review authors alongside data extraction. In order to ensure that both researchers are assessing the quality of included texts reliably, 25% of the papers will initially be extracted by both reviewers and then compared. Any inconsistencies will be thoroughly discussed. For each study, researchers will use the Newcastle-Ottawa Scale to assess methodological quality.

Strategy for data synthesis

Study characteristics will be summarised in text, and provided in table format.

Meta-analysis will be conducted if studies are similar enough to pool (through assessments for clinical and statistical heterogeneity), and there are a sufficient number of studies using similar outcome measures. If not, a narrative synthesis will be conducted.

Analysis of subgroups or subsets

If a large enough subsample is identified, subgroup analysis of different age groups may be carried out. We will consider analysis of gender, race, country of study, different types of samples e.g. college students, and symptom level at baseline.

Contact details for further information

Anna Roach

anna.roach3@nhs.net

Organisational affiliation of the review

Unit for Social and Community Psychiatry (World Health Organisation Collaborating Centre for Mental Health Service Development), Newham Centre for Mental Health, London E13 8SP

https://www.qmul.ac.uk/socialpsych/

Review team members and their organisational affiliations

Miss Anna Roach. East London NHS Trust

Ms Diliniya Sureshkumar. Unit for Social and Community Psychiatry, Queen Mary University of London

Professor Stefan Priebe. Unit for Social and Community Psychiatry, Queen Mary University of London

Type and method of review

Epidemiologic, Prognostic, Systematic review

Anticipated or actual start date

03 May 2021

Anticipated completion date

For peer review only

31 August 2021

Funding sources/sponsors

Own account

Conflicts of interest

Language

English

Country

England

Stage of review

Review Ongoing

Subject index terms status

Subject indexing assigned by CRD

Subject index terms

Adolescent; Adolescent Health; Anxiety; Anxiety Disorders; Depression; Disease Progression; Humans; Mental Disorders; Mental Health; Mental Health Recovery; Prognosis; Psychological Distress; Young Adult

Date of registration in PROSPERO

27 April 2021

Date of first submission

26 April 2021

Stage of review at time of this submission

For peer review only

Stage	Started	Completed
Preliminary searches	Yes	No
Piloting of the study selection process	No	No
Formal screening of search results against eligibility criteria	No	No
Data extraction	No	No
Risk of bias (quality) assessment	No	No
Data analysis	No	No

The record owner confirms that the information they have supplied for this submission is accurate and complete and they understand that deliberate provision of inaccurate information or omission of data may be construed as scientific misconduct.

The record owner confirms that they will update the status of the review when it is completed and will add publication details in due course.

Versions

27 April 2021

For peer review only

Search strategy:

OVID (Embase, & Global Health)

1) Longitudinal OR Cohort OR `follow up` OR outcome* OR prognos* OR prospective OR retrospective AND

2) Adolescen* OR `young people` OR youth* OR teen* OR minor* OR juvenile* OR `young person*` OR `student*` OR `high school` OR `middle school` OR `secondary school` OR child* OR `young women` OR `young men` OR `boy*` OR `girl*` AND

3) `Mental distress` OR depress* OR anxiety OR anxious OR `psychological distress` OR `distress` OR `internal* disorder` OR `internal* behavio*` AND

4) `one year` OR `12 month*` OR `1 year`

5) 1 AND 2 AND 3 AND 4

MEDLINE (via PubMed):

(Longitudinal OR Cohort OR "follow up" OR outcome* OR prognos* OR prospective OR retrospective) AND (("Adolescent/psychology"[Mesh]) OR "young people" OR youth OR teen* OR minor OR juvenile* OR "young person" OR "student*" OR "high school" OR "middle school" OR "secondary school" OR child* OR "young women" OR "young men" OR boy OR girl*) AND ("Mental distress" OR ("Depression/diagnosis"[Mesh] OR "Depression/psychology"[Mesh] OR "Depression/rehabilitation"[Mesh] OR "Depression/therapy"[Mesh]) OR ("Anxiety/diagnosis"[Mesh] OR "Anxiety/psychology"[Mesh] OR "Anxiety/rehabilitation"[Mesh] OR "Anxiety/therapy"[Mesh]) OR ("Psychological Distress/diagnosis"[Mesh] OR "Psychological Distress/psychology"[Mesh] OR "Psychological Distress/rehabilitation"[Mesh] OR "Psychological Distress/therapy"[Mesh]) OR "distress" OR "internal* disorder" OR "internal* behavio*" OR ("Emotions/diagnosis"[Mesh] OR "Emotions/psychology"[Mesh] OR "Emotions/rehabilitation"[Mesh] OR "Emotions/therapy"[Mesh])) AND ("one year" OR "12 month*" OR "1 year")

MeSH Terms used:
"depression"
"anxiety"
"emotions"
"psychological distress"
"adolescent"

For peer review only

PsycINFO

(Longitudinal OR Cohort OR “follow up” OR outcome* OR prognos* OR prospective OR retrospective) AND (Adolescen* OR "young people" OR youth OR teen* OR minor OR juvenile* OR “young person” OR “student*” OR “high school” OR “middle school” OR “secondary school” OR child* OR “young women” OR “young men” OR boy* OR girl*) AND ("Mental distress" OR depress* OR anxiety OR anxious OR “psychological distress” OR “distress” OR “internal* disorder” OR internal* behavio*) AND ("one year" OR "12 month*" OR “1 year”)

Web of Science

(Longitudinal OR Cohort OR “follow up” OR outcome* OR prognos* OR prospective OR retrospective) AND (Adolescen* OR "young people" OR youth OR teen* OR minor OR juvenile* OR “young person” OR “student*” OR “high school” OR “middle school” OR “secondary school” OR child* OR “young women” OR “young men” OR boy* OR girl*) AND ("Mental distress" OR depress* OR anxiety OR anxious OR “psychological distress” OR “distress” OR “internal* disorder” OR internal* behavio*) AND ("one year" OR "12 month*" OR “1 year”)

For peer review only

Risk of bias (quality) assessment

All the literature retrieved was critically appraised using common variables on an individual basis. Independent reviewers appraised methodological quality and risk of bias according to the Newcastle-Ottawa Scale (NOS) that has validity for use in cohort studies Wells et al. An eight-item scale with three quality parameters (i) selection, (ii) comparability and (iii) outcome. We rated the quality of the studies (good, fair, and poor) by allocating each domain with stars in this manner:

- A **Good** quality score was awarded 3 or 4 stars in selection, 1 or 2 in comparability and 2 or 3 stars in outcomes.
- A **Fair** quality score was awarded 2 stars in selection, 1 or 2 stars in comparability, and 2 or 3 stars in outcomes.
- A **Poor** quality score was awarded 0 or 1 star(s) in selection, 0 stars in comparability, and 0 or 1 star(s) in outcomes domain (Wells et al., 2000)

Newcastle-Ottawa Scale Quality Assessment of included studies (TABLE X)

Author(s)	Selection				Comparability		Outcome		
	Representativeness of cohort	Selection of non-exposed	Ascertainment of exposure	Outcome of interest not present at start	Control for age and sex	For additional factors	Assessment of outcome	Length of follow-up	Adequacy of follow up
Jinnin, R et al (2016)	*	-	*	*	**		-	*	*
Leikanger, E & Larsson, B (2012)	*	-	-	*	*		-	*	*
Goodyer, I.M & Herbert, J (2003)	*	-	*	*	**		-	*	*
Fan, F et al (2016)	*	-	*	*	*		-	*	*
Yang, H-J et al (2008)	*	-	-	*	**		-	*	*

Wells, G., Shea, B., O'Connel, D., Peterson, J, Welch, V., Loso, M., Tugwell, P. (2000). The Newcastle-ottawa scale (NOS) for assessing the quality of nonrandmoised studies in meta-analyses. Ottawa, ON, Canada: Ottawa Hospital Research Institute.

For peer review only

BMJ Open

One-year recovery rates for young people with depression and/or anxiety not receiving treatment: A systematic review and meta-analysis

Journal:	*BMJ Open*
Manuscript ID	bmjopen-2023-072093.R1
Article Type:	Original research
Date Submitted by the Author:	22-Apr-2023
Complete List of Authors:	Roach, Anna; Queen Mary University of London<br>Sureshkumar, Diliniya Stanislaus; Queen Mary University of London<br>Elliot, Kathryn; Queen Mary University of London<br>Hidalgo-Padilla, Liliana; Universidad Peruana Cayetano Heredia<br>van Loggerenberg, Francois; Youth Resilience Unit, Newham Centre for Mental Health ; Queen Mary University of London, Unit for Social and Community Psychiatry<br>Hounsell, Lauren; Queen Mary University of London<br>Jakaite, Zivile; Queen Mary University of London<br>Esnal, Fernando; Universidad de Buenos Aires<br>Donaghy, Jade; Queen Mary University of London<br>Bird, Victoria; Queen Mary University of London, Unit for Social and Community Psychiatry<br>Priebe, Stefan; Queen Mary University of London
**Primary Subject Heading**:	Mental health
Secondary Subject Heading:	Paediatrics
Keywords:	MENTAL HEALTH, Child & adolescent psychiatry < PSYCHIATRY, Depression & mood disorders < PSYCHIATRY, Anxiety disorders < PSYCHIATRY

SCHOLARONE™
Manuscripts

I, the Submitting Author has the right to grant and does grant on behalf of all authors of the Work (as defined in the below author licence), an exclusive licence and/or a non-exclusive licence for contributions from authors who are: i) UK Crown employees; ii) where BMJ has agreed a CC-BY licence shall apply, and/or iii) in accordance with the terms applicable for US Federal Government officers or employees acting as part of their official duties; on a worldwide, perpetual, irrevocable, royalty-free basis to BMJ Publishing Group Ltd ("BMJ") its licensees and where the relevant Journal is co-owned by BMJ to the co-owners of the Journal, to publish the Work in this journal and any other BMJ products and to exploit all rights, as set out in our licence.

The Submitting Author accepts and understands that any supply made under these terms is made by BMJ to the Submitting Author unless you are acting as an employee on behalf of your employer or a postgraduate student of an affiliated institution which is paying any applicable article publishing charge ("APC") for Open Access articles. Where the Submitting Author wishes to make the Work available on an Open Access basis (and intends to pay the relevant APC), the terms of reuse of such Open Access shall be governed by a Creative Commons licence – details of these licences and which Creative Commons licence will apply to this Work are set out in our licence referred to above.

Other than as permitted in any relevant BMJ Author's Self Archiving Policies, I confirm this Work has not been accepted for publication elsewhere, is not being considered for publication elsewhere and does not duplicate material already published. I confirm all authors consent to publication of this Work and authorise the granting of this licence.

For peer review only

One-year recovery rates for young people with depression and/or anxiety not receiving treatment: A systematic review and meta-analysis

Anna Roach, M.Sc[a], Diliniya Stanislaus Sureshkumar[a], Kathryn Elliot[a], Liliana Hidalgo-Padilla[b], Francois van Loggerenberg[c], Lauren Hounsell[a], Zivile Jakaite[a], Fernando Esnal[d], Jade Donaghy[a], Victoria Bird[a] and Stefan Priebe, M.S.(Psych), Ph.D[a]

[a] Unit for Social and Community Psychiatry (World Health Organization Collaborating Centre for Mental Health Services Development), Newham Centre for Mental Health, Queen Mary University of London, London, UK

[b] CRONICAS Center of Excellence in Chronic Diseases, Universidad Peruana Cayetano Heredia, Lima, Peru

[c] Youth Resilience Unit (YRU), Newham Centre for Mental Health, Queen Mary University of London, London, UK

[d] Universidad de Buenos Aires, Buenos Aries, Argentina

Address correspondence: Anna Roach, Unit of Social and Community Psychology, Newham Centre for Mental Health, Queen Mary University of London; Email address: anna.roach.21@ucl.ac.uk

Word count: 3359

For peer review only

ABSTRACT

Objectives: To systematically review one-year recovery rates for young people experiencing depression and/or anxiety who are not receiving any specific mental health treatment.

Design: Systematic review and meta-analysis

Data Sources: : MEDLINE, Embase, PsycINFO, Web of Science, and Global Health were searched for articles published from 1980 through to August 2022.

Eligibility Criteria: Articles were peer reviewed, published in English and had baseline and one-year follow up depression and/or anxiety outcomes for young people aged 10-24 years without specific treatment.

Data extraction and synthesis: Three reviewers extracted relevant data. Meta-analysis was conducted to calculate the proportion of individuals classified as recovered after one year. The quality of evidence was assessed by the Newcastle-Ottawa Scale.

Results: Of the 17,250 references screened for inclusion, five articles with 1,011 participants in total were included. Studies reported a one-year recovery rate of between 47-64%. In the meta-analysis, the overall pooled proportion of recovered young people is 0.54 (0.45 – 0.63).

Conclusions: The findings suggest that after one-year about 54% of young people with symptoms of anxiety and/or depression recover without any specific mental health treatment. Future research should identify individual characteristics predicting recovery and explore resources and activities which may help young people recover from depression and/or anxiety.

PROSPERO registration number: CRD42021251556

Data availability statement: All data relevant to the study are included in the article or uploaded as online supplemental information.

Key words: Depression, anxiety, recovery, one-year outcomes, young people

STRENGTHS AND LIMITATIONS OF THIS STUDY

- The study design made it possible to accumulate and present research across multiple studies, countries and contexts
- Thorough literature search of five major electronic databases and reporting as per Preferred Reporting Items for Systematic Reviews and Meta-Analyses guidelines.
- Evidence base with data of a total of 1,011 participants is limited, and the small number of studies meant moderator analyses could not be conducted
- No available information about how long symptoms had been present for at baseline meaning participants with very short episodes of depression and/or anxiety were likely missed

INTRODUCTION

Adolescence and young adulthood are important developmental periods of social, behavioural, and psychological change where there is a transition from the dependence of childhood to adulthood independence [1]. However, these periods are also associated with increased risk of experiencing mental distress with most functional mental disorders beginning before the age of 25 years [2].

For peer review only

Research suggests that the majority of young people are likely to experience mental distress at some point of time [3], yet the spontaneous prognosis of such distress remains uncertain. An understanding of the prognosis without treatment may inform appropriate responses on individual and public health levels [4].

Improving mental health among young people has been identified by the World Health Organisation (WHO) as a key priority to promote social and economic development [5]. Experiencing depression and anxiety in early life is associated with poor outcomes, such as high levels of distress and disability, future physical and psychiatric morbidity, and educational and social impairment [6-8]. Strengthening the resilience of young people is suggested as key to promoting long term mental health outcomes [9].

Although some research estimates that a proportion of those who experience depressive episodes in adolescence will go on to experience at least one recurrent episode in adulthood [10], to date there has been no systematic exploration of the one-year recovery outcomes of adolescent depression and/or anxiety.

Against this background, we conducted a systematic review and meta-analysis of recovery rates of young people with depression and/or generalised anxiety who did not receive any specific mental health treatment. For an inclusive approach, we used a wide age range including adolescence and young adulthood, i.e. 10-24 years, following the WHO definition of young people [11]. The one-year time frame appears relevant for considering whether young people with symptoms of depression and/or anxiety should be referred to mental health services and has the pragmatic advantage that studies tend to report outcomes for one-year rather than for other timeframes.

METHODS

We conducted a systematic review and meta-analysis to estimate the proportion of young people who recover from depression and anxiety in a one-year period. Methodology and reporting for this systematic review are consistent with PRISMA guidelines [12]. A PRISMA checklist is provided in *Supplementary Material 1*. A protocol for this review was developed a priori and registered on PROSPERO (CRD42021251556) and is available in *Supplementary Material 2*.

Search strategy

A comprehensive literature search was conducted in five electronic databases: MEDLINE, Embase, PsycINFO, Web of Science, and Global Health for articles published from 1980 through to August 2022. The search strategy was adapted for the different databases and search terms, included the study design (longitudinal, cohort, prospective, etc), terms relating to young people (adolescents, youth, school, etc), terms encompassing mental distress (depression, anxiety, internalising disorder, etc) and a one year or 12 month follow up. An example search strategy and the MeSH headings used are available in *Supplementary Material 3.* The reference and citation lists were also scanned of eligible articles to supplement our database searches.

Eligibility criteria

Articles were eligible if they included

- Participants defined by WHO as "young people" (aged 10-24) at baseline, which includes adolescents (aged 10-19) and youth (aged 15-24).

For peer review only - http://bmjopen.bmj.com/site/about/guidelines.xhtml

- A validated self-report or observer-rated measure of depression and/or generalised anxiety or a diagnosis of depression and/or generalised anxiety as defined by the ICD-10 or equivalent.
- A prospective design including one year follow-up depression and/or anxiety outcomes.

Only studies reporting individual recovery rates of anxiety and/or depression were included, studies reporting group recovery rates were not included unless the whole sample had symptoms with anxiety and/or depression at baseline. Furthermore, included articles had to be published in English in peer-reviewed journals with longitudinal cohort research study designs (with one-year follow-up data available).

We excluded all studies where young people were receiving pharmacological or psychological treatment or other specific interventions for their mental health. Grey literature was also not included.

Data collection and analysis

A large team of independent reviewers (AR, DSS, LH, FvL, ZJ, LH, FE, JD) screened titles and abstracts to ascertain potential eligibility. Those appearing relevant underwent full-text review, of which 20% were double screened (KE). The disagreement rate was 3%, and the conflicts were resolved by an independent reviewer (FvL).

Data extraction

Three reviewers (AR, DSS and KE) independently extracted data from eligible articles into a computerised extraction form developed before review (available on request). Data extracted included study objective, country/setting of research, sample size, baseline outcome measure, validated measure of depressive and/or anxiety symptoms (including if self-report/clinician led), age at baseline, follow up duration, follow up outcome, method of analysis, and covariates.

Risk of bias

The methodological quality of eligible articles were assessed using the Newcastle-Ottawa Scale (NOS) [13] a quality assessment tool used for non RCT studies including case-control and cohort studies. The NOS uses a star rating system; stars are assigned for eight items grouped into three categories assessing selection of study groups, comparability, and outcomes.

Data Analysis

A meta-analysis was conducted within STATA to calculate the proportion of individuals classified as recovered. We used Wilson's method [14] to calculate 95% confidence intervals for proportion estimates. The approach produces asymmetric confidence intervals in studies with low proportion rates. Heterogeneity among studies was estimated based on Cochran Q and reported using I^2 (and 95% confidence interval of the I^2). $I^2 > 75\%$ is considered indicative of high heterogeneity. Given the heterogeneity within the papers, we used a random effects meta-analysis applying the metan code within STATA Version 15 [15].

As it is common for meta-analyses of observational or cohort data to report high levels of heterogeneity, prediction intervals were also calculated. The prediction interval includes the plausible range of estimates we would expect the effect of a new study to be within. They are wider than confidence intervals as they include the random variation of individual measurement as well as the uncertainty of estimating the population effect size. To calculate the prediction intervals, the

For peer review only - http://bmjopen.bmj.com/site/about/guidelines.xhtml

“meta set” command was first used to declare the data as meta data, this was followed by the “meta summarize” and “predinterval” commands.

Patient and Public Involvement

No patients involved.

RESULTS

A search in five electronic databases identified 17,250 articles of which 16,200 were excluded based on the eligibility criteria *(Fig. 1, PRISMA Diagram)*. In total, five studies were included in our analysis reporting recovery data of 1,011 participants.

Many studies were excluded as they showed a general trend in recovery rather than reporting individual recovery rate. All studies included were longitudinal studies that examined the one-year outcomes of depressive and/or anxiety symptoms in young people. These papers are summarised in *Supplementary Table 1*.

Several papers did not provide the required information for this review; thus, the authors of each study were contacted to gain the necessary details. There were four authors that did not reply and therefore those papers were excluded from the analysis.

Figure 1: PRISMA flowchart of paper selection process

Study Characteristics

A total of 1,011 participants were included in this systematic review. The sample size ranged from 30 to 455 with the age at baseline varying from 12–19. Of the five papers in this review four were from high-income countries [16-19] and one from a middle-income country [20]. The majority of the studies reported the one year outcome of depression [16-20] with only one study observing the outcomes of anxiety in young people [18]. All young people met either a threshold for elevated symptoms or diagnosis of depression and/or anxiety at baseline, as defined by the authors of the respective studies. All participants in these studies were recruited from education settings [16-20].The majority of the measures used were self-reporting scales, except for the Kiddie-SADS measure which is clinician led.

Risk of bias

The quality appraisal of the five studies is presented in *Supplementary Table 1*, with three of the studies assessed to be of good quality scoring (i.e. scoring 3 or 4 stars in selection, 1 or 2 in comparability and 2 or 3 stars in outcomes) and only two studies deemed to be of fair reporting quality (i.e. scoring 2 stars in selection, 1 or 2 stars in comparability, and 2 or 3 stars in outcomes). All studies were deemed representative of the target population and controlled for various confounding variables. The five studies all stated a one-year follow up period with little to no follow-up loss. Three studies administered structured interviews to ascertain the exposure; however, none of the studies used a formal assessment to determine the outcome as they relied upon the use of self-reported measures *(see Supplementary material 4 for full quality appraisal)*.

For peer review only - http://bmjopen.bmj.com/site/about/guidelines.xhtml

One-year recovery

Five studies provided data for the meta-analysis *(see Figure 2)*. The overall pooled proportion of young people to recover (defined as symptom reduction or remission) is 0.54 (0.45 – 0.63), which indicates roughly half (54%) of young people recover without any specific mental health treatment or intervention within a year. Although there is substantial heterogeneity across the eligible studies (I^2=84%), to account for the high heterogeneity evident in meta-analyses of observation data, prediction intervals were also calculated (90% PI 0.340, 0.738), to estimate an interval range in which a future observation will fall.

Figure 2: Forest plot showing pooled proportions and 95% confidence intervals (CIs) for recovery rate and symptoms reduction of anxiety and/or depression for young people, after 1 year.

All five studies reported a recovery rate ranging from 47% to 64%. There was only one study that looked at the recovery of symptoms of anxiety and they found that 48% of young people (95% CI 0.39-0.56) had gone from scoring high on the SCARED scale to low-moderate after a year [18].

Gender differences for recovery of young people with depression were reported in only one study [18], who reported that 67 adolescents (47.5 % of the high scorers at baseline: 47.8 % of girls, and 46.4 % of boys) had high scores at baseline but low to moderate scores at 12-month follow up.

DISCUSSION

The findings of this systematic review and meta-analysis indicate that 54% of young people with symptoms of depression and/or anxiety tend to achieve recovery, defined as symptom reduction below a threshold within a year, when they do not receive specific mental health treatment. The included studies show relatively consistent results with limited variation. Based on the available evidence, the most likely spontaneous prognosis for young people with symptoms of depression and/or anxiety is that about half of them will have recovered after a year. Such process can be understood as a sign of resilience with young people bouncing back from their experience of distress [21].

There is no systematic review in adults that offers a direct comparison, however literature suggests that minor depression is likely to persist and poses an elevated risk of worsening over one year in 60% of older adults [22].

Strengths and limitations

This is the first systematic review and meta-analysis to explore one-year recovery rates of young people with depression and/or anxiety. Symptom reduction across depression and anxiety was shown in similar proportions across the different studies, with markedly small margins which suggest relatively robust findings. Pooled analyses were therefore representative of papers and one study with a larger sample size was not skewing the results. Finally, most articles included were of moderate to high methodologic quality and used validated measures with strong psychometric properties. Previous research suggests adolescents are capable of providing valid self-reports of depressive symptoms [23], strengthening the reliability of our findings.

This review does however have several limitations. Firstly, we have no systematic information about how long symptoms had been present for at baseline. Studies started with cross-sectional identification of participants who met the symptom levels defined as inclusion criteria at the time of recruitment to the cohort study. These studies were likely to miss participants with very short episodes of depression and/or anxiety and recruit participants whose symptoms had already been

For peer review only - http://bmjopen.bmj.com/site/about/guidelines.xhtml

there for longer. Thus, the spontaneous prognosis of young people experiencing their first symptoms of depression and/or anxiety is likely to be more favourable than the 54% in this review. Secondly, given the world-wide interest in the mental health of young people, the evidence base with data of a total of 1,011 participants is limited, and there was only one study exploring recovery rates of anxiety. Most research studies are conducted in clinical settings where young people are receiving treatment.

It was not possible to access data from the control groups or "treatment as usual" groups from randomised controlled trials, despite requesting this information from authors, furthermore in trials conducted in clinical services it would be unlikely that the control group are not receiving any intervention. Due to the small number of included studies, moderation analysis on patient factors such as age and gender could not be conducted. Thirdly, we focused on only two time points, i.e. baseline and one-year follow-up, and did not consider what happened in between the two time points or after the follow-up. For instance, participants who had symptoms after a year might have been symptom-free in between, and participants we regarded as recovered may have relapsed after the follow-up. The findings do therefore not imply that 54% will remain recovered in the future. Although effort was made to include young people not receiving treatment, it is possible that some were accessing support outside of clinical services and the context of this study. Finally, the studies included in this review are highly heterogeneous. Although this suggests that similar recovery rates can be found in very different contexts, it may also challenge whether it is appropriate to derive an overall estimate from that set of studies. The data was also not sufficient to distinguish between narrower age bands within the wide group of young people.

Implications

Although there is evidence that adolescents with depression go on to experience episodes of depression in later life [24] our review suggests that significant symptom reduction occurs after one year in at least half of young people who are affected. Three to nine percent of teenagers meet the criteria for depression at any one time [25], and the question arises as to whether they should routinely be considered for specialised treatments or whether one should wait with such decisions for a year by which time about 54% are likely to have recovered without treatment.

A recent systematic review found that although psychotherapies for depression in young people can be effective, more than 60% of those receiving therapy do not respond [26]. Similarly, antidepressants have been found to have only a small therapeutic effect in this group [27]. The decision as to whether specific treatments should be considered is further complicated by the absence of evidence-based predictor variables for a) who would or would not recover without treatment, and b) who would or would not benefit from specific treatments. School-based and community programs are gaining traction, especially following the COVID-19 pandemic [28] but further studies are needed to demonstrate long term outcomes and successful implementation into routine practice.

It is important to analyse and further understand why more than half of young people recover, while other young people continue to report symptoms of depression and anxiety. Further research should identify personal characteristics predicting recovery. Since the predictive value of common socio-demographic characteristics is likely to be limited, such research may have to consider individual experiences, attitudes, family cohesion and community links.

The main task for future research however may be to explore what personal, family and community resources young people can find and utilise that help them recover from depression and anxiety.

For peer review only - http://bmjopen.bmj.com/site/about/guidelines.xhtml

Such research may lead to social and public health interventions that can make a difference to large groups of affected young people and substantially improve the recovery rate of 54% found in this review.

Given the relatively small variability of recovery rates in this review, future intervention studies may use the 54% finding of this study as a yardstick to evaluate outcomes, when recruiting and assessing control groups is impractical or impossible.

CONCLUSION

In conclusion, although it is encouraging to find that half of young people tend to recover without treatment, if almost 50% still have symptoms after one year, concerns remain. This is especially so as previous research shows only a small effect of both pharmacological and psychological interventions, although such treatments through mental health professionals generate significant costs. This calls for further development and evaluation of treatments in mental health care, but also interventions delivered within families, schools, and communities that prevent mental distress in young people as far as possible and help those experiencing symptoms of depression or anxiety to recover.

Contributor statement: AR and SP came up with the research question. AR, DS and LH-P led data extraction with KE, FvL, LH, ZJ, FE and JD assisting with title and abstract screening and then full text screening. AR, DS, KE and VB analysed the included papers. AR and DS drafted the manuscript and all provided feedback on the paper before submission.

Competing interests: The authors declared no potential conflicts of interest with respect to the research, authorship, and/or the publication of this article.

Funding: This research did not receive any specific grant from funding agencies in the public, commercial, or not-for-profit sectors.

Data sharing statement: All data relevant to the study are included in the article or uploaded as online supplemental information.

Ethics approval statement: Ethical approval was not required for this systematic review and meta-analysis.

For peer review only - http://bmjopen.bmj.com/site/about/guidelines.xhtml

For peer review only

References

1. Priebe S, Fung C, Brusco LI, et al. Which resources help young people to prevent and overcome mental distress in deprived urban areas in Latin America? A protocol for a prospective cohort study. BMJ open 2021;**11**(9):e052339.
2. Solmi M, Radua J, Olivola M, et al. Age at onset of mental disorders worldwide: large-scale meta-analysis of 192 epidemiological studies. Molecular psychiatry 2022;**27**(1):281-95.
3. Kessler RC, Amminger GP, Aguilar-Gaxiola S, Alonso J, Lee S, Ustun TB. Age of onset of mental disorders: a review of recent literature. Current opinion in psychiatry 2007;**20**(4):359.
4. Patton GC, Coffey C, Romaniuk H, et al. The prognosis of common mental disorders in adolescents: a 14-year prospective cohort study. The Lancet 2014;**383**(9926):1404-11.
5. Varese F, Smeets F, Drukker M, et al. Childhood adversities increase the risk of psychosis: a meta-analysis of patient-control, prospective-and cross-sectional cohort studies. Schizophrenia bulletin 2012;**38**(4):661-71.
6. Fogel J, Eaton W, Ford D. Minor depression as a predictor of the first onset of major depressive disorder over a 15-year follow-up. Acta Psychiatrica Scandinavica 2006;**113**(1):36-43.
7. Goodman E, Huang B. Socioeconomic status, depressive symptoms, and adolescent substance use. Archives of pediatrics & adolescent medicine 2002;**156**(5):448-53.
8. Merikangas KR, He J-p, Burstein M, et al. Lifetime prevalence of mental disorders in US adolescents: results from the National Comorbidity Survey Replication–Adolescent Supplement (NCS-A). Journal of the American Academy of Child & Adolescent Psychiatry 2010;**49**(10):980-89.
9. Anderson K, Priebe S. Concepts of resilience in adolescent mental health research. Journal of Adolescent Health 2021;**69**(5):689-95.
10. Johnson D, Dupuis G, Piche J, Clayborne Z, Colman I. Adult mental health outcomes of adolescent depression: A systematic review. Depression and anxiety 2018;**35**(8):700-16.
11. WHO. Adolescent Health. Secondary Adolescent Health 2021. https://www.who.int/southeastasia/health-topics/adolescent-health.
12. Liberati A, Altman DG, Tetzlaff J, et al. The PRISMA statement for reporting systematic reviews and meta-analyses of studies that evaluate health care interventions: explanation and elaboration. Journal of clinical epidemiology 2009;**62**(10):e1-e34.
13. Wells GA, Shea B, O'Connell D, et al. The Newcastle-Ottawa Scale (NOS) for assessing the quality of nonrandomised studies in meta-analyses: Oxford, 2000.
14. Wilson EB. Probable inference, the law of succession, and statistical inference. Journal of the American Statistical Association 1927;**22**(158):209-12.
15. Stata Statistical Software: Release 15 [program]. TX: StataCorp LLC., 2017.
16. Fan F, Zhou Y, Liu X. Sleep disturbance predicts posttraumatic stress disorder and depressive symptoms: a cohort study of Chinese adolescents. The Journal of clinical psychiatry 2017;**78**(7):19701.
17. Goodyer I, Herbert J, Tamplin A. Psychoendocrine antecedents of persistent first-episode major depression in adolescents: a community-based longitudinal enquiry. Psychological medicine 2003;**33**(4):601-10.
18. Leikanger E, Larsson B. One-year stability, change and incidence in anxiety symptoms among early adolescents in the general population. European Child & Adolescent Psychiatry 2012;**21**(9):493-501.
19. Jinnin R, Okamoto Y, Takagaki K, et al. Detailed course of depressive symptoms and risk for developing depression in late adolescents with subthreshold depression: a cohort study. Neuropsychiatric Disease and Treatment 2017;**13**:25.

For peer review only - http://bmjopen.bmj.com/site/about/guidelines.xhtml

For peer review only

20. Yang H-J, Chiu Y-J, Soong W-T, Chen WJ. The roles of personality traits and negative life events on the episodes of depressive symptoms in nonreferred adolescents: a 1-year follow-up study. Journal of Adolescent Health 2008;**42**(4):378-85.
21. Windle G. What is resilience? A review and concept analysis. Reviews in clinical gerontology 2011;**21**(2):152-69.
22. Lyness JM, Chapman BP, McGriff J, Drayer R, Duberstein PR. One-year outcomes of minor and subsyndromal depression in older primary care patients. International Psychogeriatrics 2009;**21**(1):60-68.
23. Moretti M, Fine S, Haley G, Marriage K. Childhood and adolescent depression: Child-report versus parent-report information. Journal of the American Academy of Child Psychiatry 1985;**24**(3):298-302.
24. Clayborne ZM, Varin M, Colman I. Systematic review and meta-analysis: adolescent depression and long-term psychosocial outcomes. Journal of the American Academy of Child & Adolescent Psychiatry 2019;**58**(1):72-79.
25. Bansal V, Goyal S, Srivastava K. Study of prevalence of depression in adolescent students of a public school. Industrial psychiatry journal 2009;**18**(1):43.
26. Cuijpers P, Karyotaki E, Ciharova M, et al. The effects of psychological treatments of depression in children and adolescents on response, reliable change, and deterioration: a systematic review and meta-analysis. European Child & Adolescent Psychiatry 2021:1-16.
27. Vitiello B, Ordóñez AE. Pharmacological treatment of children and adolescents with depression. Expert opinion on pharmacotherapy 2016;**17**(17):2273-79.
28. Hamoda HM, Chiumento A, Alonge O, et al. Addressing the consequences of the COVID-19 lockdown for children’s mental health: Investing in school mental health programs. Psychiatric services 2021;**72**(6):729-31.

For peer review only

For peer review only - http://bmjopen.bmj.com/site/about/guidelines.xhtml

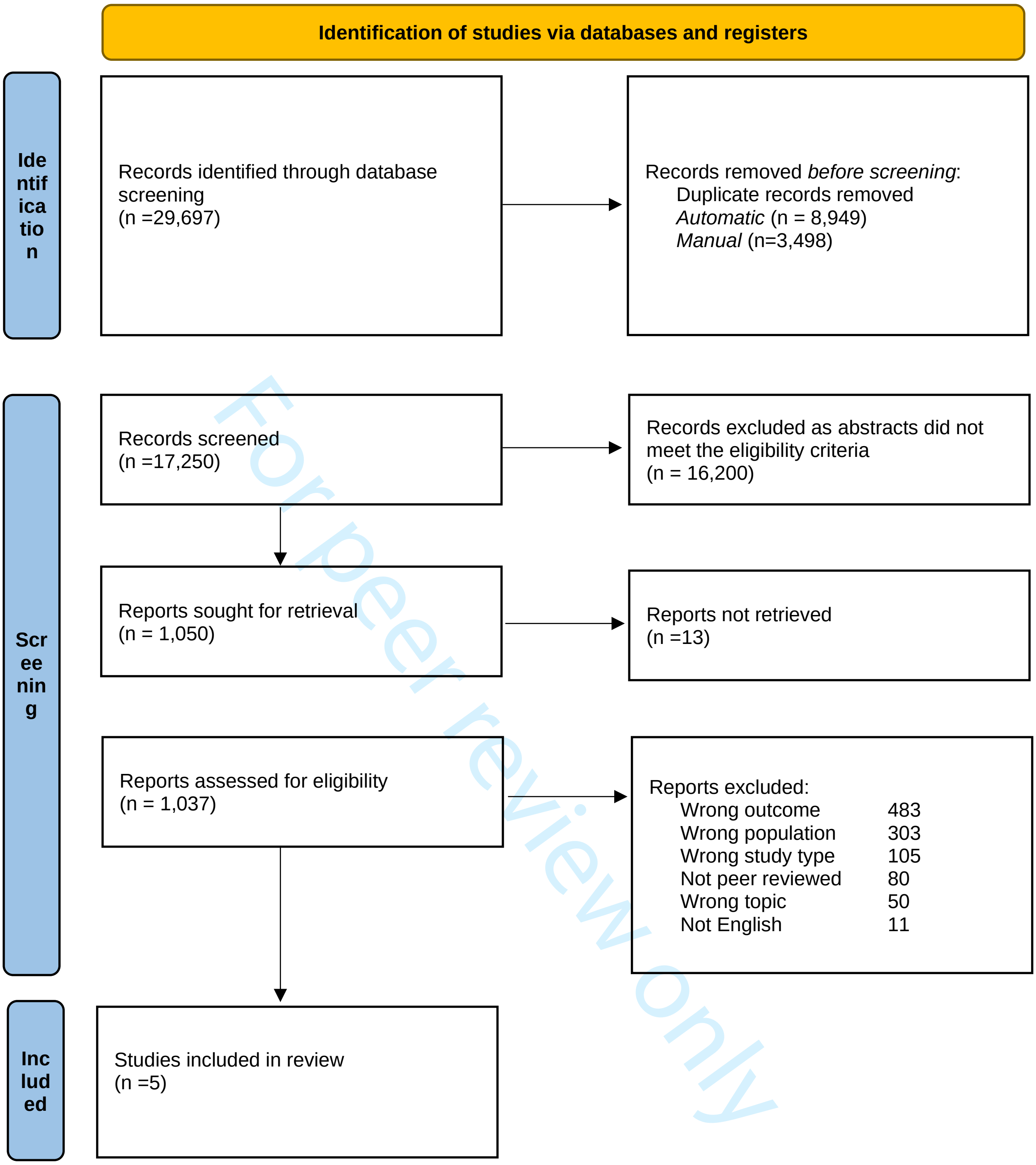

Figure 1 – PRISMA flowchart of paper selection process

For peer review only

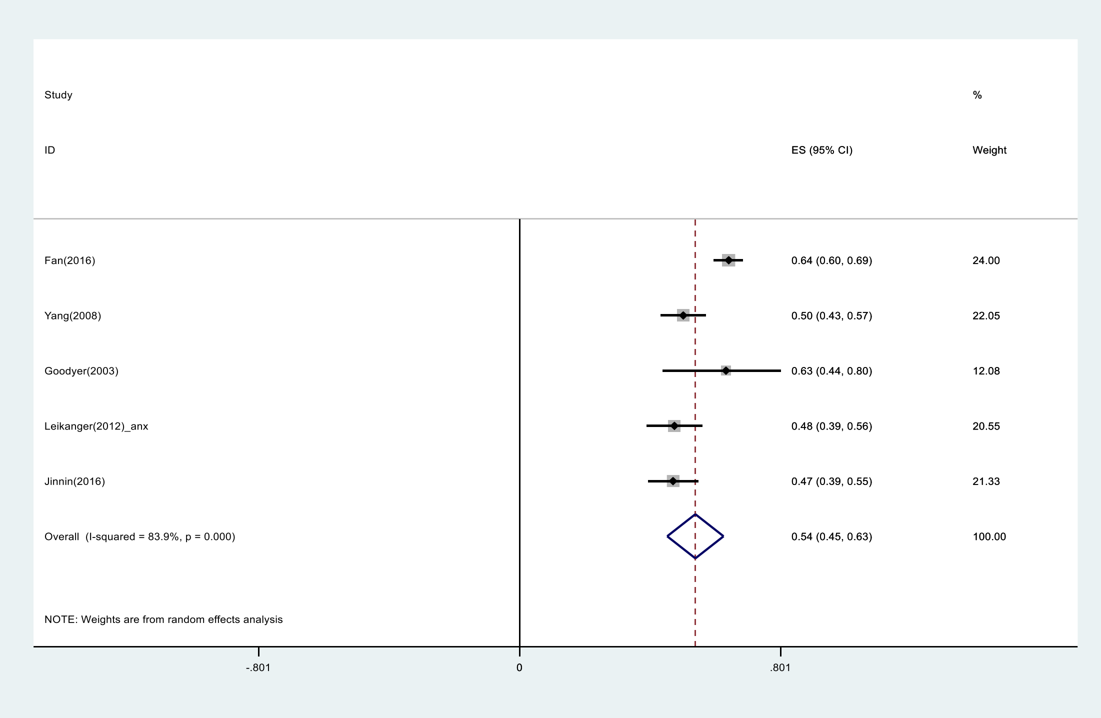

Figure 2 - Forest plot showing pooled proportions and 95% confidence intervals (CIs) for recovery rate and symptoms reduction of anxiety and/or depression for young people, after 1 year.

Author, year	Country	Setting and Population	Study Aim	Age at baseline	Sample size at baseline	Measure of depressive and/or anxiety symptoms	Cut off scores	One year follow-up	Quality Assessment (NOS)
Fan et al., 2016	China	High Schools in Dujiangyan, China; Population drawn from adolescents exposed to the 2008 Wenchuan earthquake in China.	This study explored the associations between posttraumatic stress disorder, depressive symptoms, and sleep disturbance after the Wenchuan earthquake	13 to 17 years old	455 participants	Chinese version of the Depression Self-Rating Scale for Children *(Self-report)*	Score of 15 has been used as the cut-off for screening depressive disorders	292 participants recovered from depressive symptoms (64%)	6
Yang et al., 2008	Taiwan	Middle Schools in Taiwan; population drawn from the students	This study evaluated the influence of personality traits and non-severe life events on depressive episodes in adolescents from six middle schools	12 to 16 years old	213 participants	Chinese version of the Centre for Epidemiologic Studies Depression *(Self-report)*	Wave 2 CES-D score becoming 28 or less by a decrease from Wave 1 CES-D score of >28	107 participants recovered (50%)	6
Goodyer et al., 2003	U.K	Three secondary schools within Cambridge; population drawn from secondary school students who met the diagnostic criteria for major depressive disorder.	This study investigated whether hormones influence recovery in a community sample of adolescents with major depression	12 to 16 years old	30 participants (depressive symptoms)	Kiddie-SADS Schedule for Schizophrenia and Affective Disorders; Mood *(Clinician led scale)* Mood and Feelings Questionnaire *(Self-report)*	-	19 participants recovered from depression (63%)	7
Leikanger, E & Larrson, B., 2012	Norway	18 schools across 17 municipalities in Sunnmøre county in western Norway;	This study explored the stability, incidences and changes in the DSM-IV related anxiety	13 to 15 years old	141 participants	The Screen for Child Anxiety Related Emotional Disorders (SCARED) Scale	Those who scored lower than 25 at baseline were defined as ‘‘low-moderate scorers’’, and those who scored 25 or	67 participants had reduced symptoms of anxiety (48%)	5

		population drawn from Norwegian adolescents	symptoms amongst Norwegian adolescents			*(Self-report)*	higher were defined as ‘‘high scorers’’		
Jinnin et al., 2016	Japan	Hiroshima University in Japan; population drawn from first-year undergraduate students	This study looked at the progression of depressive symptoms and the potential risk for developing major depressive episodes in older adolescents	18 to 19 years old	172 participants	Japanese version of BDI-II<br><br>*(Self-report)*	Low-symptom group: BDI-II score ≤ 10<br><br>Middle-symptom group: BDI-II score = 11–17<br><br>High-symptom group: BDI-II score ≥ 18	81 participants had reduced symptoms of depression (47%)	7

Table 1. Characteristics of the included studies.

For peer review only

PRISMA 2020 Checklist

Section and Topic	Item #	Checklist item	Location where item is reported
**TITLE**			
Title	1	Identify the report as a systematic review.	P1
**ABSTRACT**			
Abstract	2	See the PRISMA 2020 for Abstracts checklist.	P1
**INTRODUCTION**			
Rationale	3	Describe the rationale for the review in the context of existing knowledge.	P2/3
Objectives	4	Provide an explicit statement of the objective(s) or question(s) the review addresses.	P2/3
**METHODS**			
Eligibility criteria	5	Specify the inclusion and exclusion criteria for the review and how studies were grouped for the syntheses.	P3/4
Information sources	6	Specify all databases, registers, websites, organisations, reference lists and other sources searched or consulted to identify studies. Specify the date when each source was last searched or consulted.	P3
Search strategy	7	Present the full search strategies for all databases, registers and websites, including any filters and limits used.	Suppl2
Selection process	8	Specify the methods used to decide whether a study met the inclusion criteria of the review, including how many reviewers screened each record and each report retrieved, whether they worked independently, and if applicable, details of automation tools used in the process.	P3
Data collection process	9	Specify the methods used to collect data from reports, including how many reviewers collected data from each report, whether they worked independently, any processes for obtaining or confirming data from study investigators, and if applicable, details of automation tools used in the process.	P3/4
Data items	10a	List and define all outcomes for which data were sought. Specify whether all results that were compatible with each outcome domain in each study were sought (e.g. for all measures, time points, analyses), and if not, the methods used to decide which results to collect.	P5
	10b	List and define all other variables for which data were sought (e.g. participant and intervention characteristics, funding sources). Describe any assumptions made about any missing or unclear information.	NA
Study risk of bias assessment	11	Specify the methods used to assess risk of bias in the included studies, including details of the tool(s) used, how many reviewers assessed each study and whether they worked independently, and if applicable, details of automation tools used in the process.	P4, P9 Suppl3
Effect measures	12	Specify for each outcome the effect measure(s) (e.g. risk ratio, mean difference) used in the synthesis or presentation of results.	P5 & Table 1
Synthesis methods	13a	Describe the processes used to decide which studies were eligible for each synthesis (e.g. tabulating the study intervention characteristics and comparing against the planned groups for each synthesis (item #5)).	P5 & Table 1
	13b	Describe any methods required to prepare the data for presentation or synthesis, such as handling of missing summary statistics, or data conversions.	P7
	13c	Describe any methods used to tabulate or visually display results of individual studies and syntheses.	P7 & Table 1
	13d	Describe any methods used to synthesize results and provide a rationale for the choice(s). If meta-analysis was performed, describe the model(s), method(s) to identify the presence and extent of statistical heterogeneity, and software package(s) used.	P7
	13e	Describe any methods used to explore possible causes of heterogeneity among study results (e.g. subgroup analysis, meta-regression).	P7
	13f	Describe any sensitivity analyses conducted to assess robustness of the synthesized results.	NA
Reporting bias assessment	14	Describe any methods used to assess risk of bias due to missing results in a synthesis (arising from reporting biases).	NA

For peer review only - http://bmjopen.bmj.com/site/about/guidelines.xhtml

PRISMA 2020 Checklist

Section and Topic	Item #	Checklist item	Location where item is reported
Certainty assessment	15	Describe any methods used to assess certainty (or confidence) in the body of evidence for an outcome.	P7
**RESULTS**			
Study selection	16a	Describe the results of the search and selection process, from the number of records identified in the search to the number of studies included in the review, ideally using a flow diagram.	Figure 1
	16b	Cite studies that might appear to meet the inclusion criteria, but which were excluded, and explain why they were excluded.	P4
Study characteristics	17	Cite each included study and present its characteristics.	Table 1
Risk of bias in studies	18	Present assessments of risk of bias for each included study.	Table 1
Results of individual studies	19	For all outcomes, present, for each study: (a) summary statistics for each group (where appropriate) and (b) an effect estimate and its precision (e.g. confidence/credible interval), ideally using structured tables or plots.	Table 1
Results of syntheses	20a	For each synthesis, briefly summarise the characteristics and risk of bias among contributing studies.	Table 1
	20b	Present results of all statistical syntheses conducted. If meta-analysis was done, present for each the summary estimate and its precision (e.g. confidence/credible interval) and measures of statistical heterogeneity. If comparing groups, describe the direction of the effect.	Figure 2
	20c	Present results of all investigations of possible causes of heterogeneity among study results.	NA
	20d	Present results of all sensitivity analyses conducted to assess the robustness of the synthesized results.	NA
Reporting biases	21	Present assessments of risk of bias due to missing results (arising from reporting biases) for each synthesis assessed.	NA
Certainty of evidence	22	Present assessments of certainty (or confidence) in the body of evidence for each outcome assessed.	NA
**DISCUSSION**			
Discussion	23a	Provide a general interpretation of the results in the context of other evidence.	P11-13
	23b	Discuss any limitations of the evidence included in the review.	P11
	23c	Discuss any limitations of the review processes used.	P11
	23d	Discuss implications of the results for practice, policy, and future research.	P12-13
**OTHER INFORMATION**			
Registration and protocol	24a	Provide registration information for the review, including register name and registration number, or state that the review was not registered.	P1&3
	24b	Indicate where the review protocol can be accessed, or state that a protocol was not prepared.	P1&3
	24c	Describe and explain any amendments to information provided at registration or in the protocol.	NA
Support	25	Describe sources of financial or non-financial support for the review, and the role of the funders or sponsors in the review.	NA
Competing interests	26	Declare any competing interests of review authors.	NA
Availability of data, code and other materials	27	Report which of the following are publicly available and where they can be found: template data collection forms; data extracted from included studies; data used for all analyses; analytic code; any other materials used in the review.	NA

For peer review only

PRISMA 2020 Checklist

From: Page MJ, McKenzie JE, Bossuyt PM, Boutron I, Hoffmann TC, Mulrow CD, et al. The PRISMA 2020 statement: an updated guideline for reporting systematic reviews. BMJ 2021;372:n71. doi: 10.1136/bmj.n71

For more information, visit: http://www.prisma-statement.org/

For peer review only

PROSPERO
International prospective register of systematic reviews

One year outcomes for young people with depression and/or anxiety: a systematic review

Citation

Anna Roach, Diliniya Sureshkumar, Stefan Priebe. One year outcomes for young people with depression and/or anxiety: a systematic review. PROSPERO 2021 CRD42021251556 Available from: https://www.crd.york.ac.uk/prospero/display_record.php?ID=CRD42021251556

Review question

What are the one year outcomes for young people with depression and/or anxiety?

Searches

Five databases will be searched: MEDLINE via PubMed, PsycINFO, Web of Science, Embase, Global Health.

MeSH terms to be used: "depression" "anxiety" "emotions" "psychological distress" "adolescent".

Inclusion:

• Longitudinal cohort research studies (with one year follow-up data);

• Texts must include primary data on anxiety and/or depression.

Exclusion:

• Studies of a specific clinical intervention/treatment;

• Grey literature (due to lack of peer-review).

The search will be re-run prior to submission.

Types of study to be included

Inclusion:

• Longitudinal cohort research studies (with one year follow-up data);

• Texts must include primary data on anxiety and/or depression.

Studies from clinical and non-clinical contexts will be included.

Research from any country will be included.

Exclusion:

• Studies of a specific clinical intervention/treatment;

For peer review only

• Grey literature (due to lack of peer-review).

Condition or domain being studied

Depression and/or anxiety in young people.

Participants/population

Inclusion:

- Participants defined by WHO as “young people” (aged 10-24) at baseline. This includes adolescents (aged 10-19) and youth (aged 15-24);

- Young people with baseline symptom levels above a specified clinical cut off point and/or a diagnosis of depression and/or anxiety as defined by the ICD-10 or equivalent. This will be indicated by a validated self-report or observer-rated questionnaire or interview.

Exclusion:

- None.

Intervention(s), exposure(s)

The focus of this review is assessing one year outcomes for young people experiencing depression and/or anxiety.

Comparator(s)/control

Not applicable.

Context

Main outcome(s)

Main outcome: recovery rate.

Secondary outcome: symptom level change.

Additional outcome(s)

Measures of quality of life and school and work performance, and social functioning will be included in our analysis of secondary outcomes.

Data extraction (selection and coding)

A minimum of two researchers will be involved in the screening, appraisal, and data extraction. Titles and abstracts will initially be screened by one author and duplicates removed. A second review author will independently screen 25% of these to check for consistency.

The full texts of potentially eligible studies will then be retrieved and assessed for eligibility by one author, and a second researcher will screen a random subset of 25% of potentially eligible studies. Any disagreements will be resolved by discussion with a third author.

MS Excel will be used to create a data extraction table. Extracted information will include study objective,

For peer review only - http://bmjopen.bmj.com/site/about/guidelines.xhtml

country/setting of research, sample size, baseline outcome measure, validated measure of depressive and/or anxious symptoms (including if self-report/clinician led), age at baseline, follow up duration, follow up outcome, method of analysis, covariates and notes.

Risk of bias (quality) assessment

Quality assessment will be conducted by a minimum of two review authors alongside data extraction. In order to ensure that both researchers are assessing the quality of included texts reliably, 25% of the papers will initially be extracted by both reviewers and then compared. Any inconsistencies will be thoroughly discussed. For each study, researchers will use the Newcastle-Ottawa Scale to assess methodological quality.

Strategy for data synthesis

Study characteristics will be summarised in text, and provided in table format.

Meta-analysis will be conducted if studies are similar enough to pool (through assessments for clinical and statistical heterogeneity), and there are a sufficient number of studies using similar outcome measures. If not, a narrative synthesis will be conducted.

Analysis of subgroups or subsets

If a large enough subsample is identified, subgroup analysis of different age groups may be carried out. We will consider analysis of gender, race, country of study, different types of samples e.g. college students, and symptom level at baseline.

Contact details for further information

Anna Roach

anna.roach3@nhs.net

Organisational affiliation of the review

Unit for Social and Community Psychiatry (World Health Organisation Collaborating Centre for Mental Health Service Development), Newham Centre for Mental Health, London E13 8SP

https://www.qmul.ac.uk/socialpsych/

Review team members and their organisational affiliations

Miss Anna Roach. East London NHS Trust

Ms Diliniya Sureshkumar. Unit for Social and Community Psychiatry, Queen Mary University of London

Professor Stefan Priebe. Unit for Social and Community Psychiatry, Queen Mary University of London

Type and method of review

Epidemiologic, Prognostic, Systematic review

Anticipated or actual start date

03 May 2021

Anticipated completion date

For peer review only

31 August 2021

Funding sources/sponsors

Own account

Conflicts of interest

Language

English

Country

England

Stage of review

Review Ongoing

Subject index terms status

Subject indexing assigned by CRD

Subject index terms

Adolescent; Adolescent Health; Anxiety; Anxiety Disorders; Depression; Disease Progression; Humans; Mental Disorders; Mental Health; Mental Health Recovery; Prognosis; Psychological Distress; Young Adult

Date of registration in PROSPERO

27 April 2021

Date of first submission

26 April 2021

Stage of review at time of this submission

For peer review only

NIHR | National Institute for Health Research

PROSPERO
International prospective register of systematic reviews

Stage	Started	Completed
Preliminary searches	Yes	No
Piloting of the study selection process	No	No
Formal screening of search results against eligibility criteria	No	No
Data extraction	No	No
Risk of bias (quality) assessment	No	No
Data analysis	No	No

The record owner confirms that the information they have supplied for this submission is accurate and complete and they understand that deliberate provision of inaccurate information or omission of data may be construed as scientific misconduct.

The record owner confirms that they will update the status of the review when it is completed and will add publication details in due course.

Versions

27 April 2021

For peer review only

Search strategy:

OVID (Embase, & Global Health)

1) Longitudinal OR Cohort OR `follow up` OR outcome* OR prognos* OR prospective OR retrospective AND

2) Adolescen* OR `young people` OR youth* OR teen* OR minor* OR juvenile* OR `young person*` OR `student*` OR `high school` OR `middle school` OR `secondary school` OR child* OR `young women` OR `young men` OR `boy*` OR `girl*` AND

3) `Mental distress` OR depress* OR anxiety OR anxious OR `psychological distress` OR `distress` OR `internal* disorder` OR `internal* behavio*` AND

4) `one year` OR `12 month*` OR `1 year`

5) 1 AND 2 AND 3 AND 4

MEDLINE (via PubMed):

(Longitudinal OR Cohort OR "follow up" OR outcome* OR prognos* OR prospective OR retrospective) AND (("Adolescent/psychology"[Mesh]) OR "young people" OR youth OR teen* OR minor OR juvenile* OR "young person" OR "student*" OR "high school" OR "middle school" OR "secondary school" OR child* OR "young women" OR "young men" OR boy OR girl*) AND ("Mental distress" OR ("Depression/diagnosis"[Mesh] OR "Depression/psychology"[Mesh] OR "Depression/rehabilitation"[Mesh] OR "Depression/therapy"[Mesh]) OR ("Anxiety/diagnosis"[Mesh] OR "Anxiety/psychology"[Mesh] OR "Anxiety/rehabilitation"[Mesh] OR "Anxiety/therapy"[Mesh]) OR ("Psychological Distress/diagnosis"[Mesh] OR "Psychological Distress/psychology"[Mesh] OR "Psychological Distress/rehabilitation"[Mesh] OR "Psychological Distress/therapy"[Mesh]) OR "distress" OR "internal* disorder" OR "internal* behavio*" OR ("Emotions/diagnosis"[Mesh] OR "Emotions/psychology"[Mesh] OR "Emotions/rehabilitation"[Mesh] OR "Emotions/therapy"[Mesh])) AND ("one year" OR "12 month*" OR "1 year")

MeSH Terms used:
"depression"
"anxiety"
"emotions"
"psychological distress"
"adolescent"

For peer review only

PsycINFO

(Longitudinal OR Cohort OR “follow up” OR outcome* OR prognos* OR prospective OR retrospective) AND (Adolescen* OR "young people" OR youth OR teen* OR minor OR juvenile* OR “young person” OR “student*” OR “high school” OR “middle school” OR “secondary school” OR child* OR “young women” OR “young men” OR boy* OR girl*) AND ("Mental distress" OR depress* OR anxiety OR anxious OR “psychological distress” OR “distress” OR “internal* disorder” OR internal* behavio*) AND ("one year" OR "12 month*" OR “1 year”)

Web of Science

(Longitudinal OR Cohort OR “follow up” OR outcome* OR prognos* OR prospective OR retrospective) AND (Adolescen* OR "young people" OR youth OR teen* OR minor OR juvenile* OR “young person” OR “student*” OR “high school” OR “middle school” OR “secondary school” OR child* OR “young women” OR “young men” OR boy* OR girl*) AND ("Mental distress" OR depress* OR anxiety OR anxious OR “psychological distress” OR “distress” OR “internal* disorder” OR internal* behavio*) AND ("one year" OR "12 month*" OR “1 year”)

For peer review only

__Risk of bias (quality) assessment__

All the literature retrieved was critically appraised using common variables on an individual basis. Independent reviewers appraised methodological quality and risk of bias according to the Newcastle-Ottawa Scale (NOS) that has validity for use in cohort studies Wells et al. An eight-item scale with three quality parameters (i) selection, (ii) comparability and (iii) outcome. We rated the quality of the studies (good, fair, and poor) by allocating each domain with stars in this manner:

- A **Good** quality score was awarded 3 or 4 stars in selection, 1 or 2 in comparability and 2 or 3 stars in outcomes.
- A **Fair** quality score was awarded 2 stars in selection, 1 or 2 stars in comparability, and 2 or 3 stars in outcomes.
- A **Poor** quality score was awarded 0 or 1 star(s) in selection, 0 stars in comparability, and 0 or 1 star(s) in outcomes domain (Wells et al., 2000)

Newcastle-Ottawa Scale Quality Assessment of included studies (TABLE X)

**Author(s)**	**Selection**				**Comparability**		**Outcome**		
	**Representativeness of cohort**	**Selection of non-exposed**	**Ascertainment of exposure**	**Outcome of interest not present at start**	**Control for age and sex**	**For additional factors**	**Assessment of outcome**	**Length of follow-up**	**Adequacy of follow up**
Jinnin, R et al (2016)	*	-	*	*	**		-	*	*
Leikanger, E & Larsson, B (2012)	*	-	-	*	*		-	*	*
Goodyer, I.M & Herbert, J (2003)	*	-	*	*	**		-	*	*
Fan, F et al (2016)	*	-	*	*	*		-	*	*
Yang, H-J et al (2008)	*	-	-	*	**		-	*	*

Wells, G., Shea, B., O'Connel, D., Peterson, J, Welch, V., Loso, M., Tugwell, P. (2000). The Newcastle-ottawa scale (NOS) for assessing the quality of nonrandmoised studies in meta-analyses. Ottawa, ON, Canada: Ottawa Hospital Research Institute.

For peer review only
